# Isocitrate Dehydrogenase‐Mutant Astrocytomas: Risk Stratification and Therapeutic Advance

**DOI:** 10.1002/mco2.70686

**Published:** 2026-03-26

**Authors:** Shepeng Wei, Xuxu Xu, Jing Bao, Zhenjiang Pan

**Affiliations:** ^1^ Department of Neurosurgery, Shidong Hospital, University of Shanghai for Science and Technology Shanghai China

**Keywords:** isocitrate dehydrogenase‐mutant astrocytomas, molecular diagnostics, radiation therapy, surgical resection, vorasidenib

## Abstract

Isocitrate dehydrogenase (IDH)–mutant astrocytomas are recognized as a single molecular entity spanning CNS WHO Grades 2–4, and clinical behavior is shaped by early lineage‐defining alterations (IDH1/2, ATRX, TP53) and by later events linked to malignant transformation (e.g., CDKN2A/B homozygous deletion). Despite integrated grading, substantial prognostic heterogeneity is observed, and treatment decisions are increasingly informed by multidomain risk stratification rather than grade alone. In this review, contemporary molecular classification and diagnostic principles are summarized, and pragmatic risk models integrating clinical factors, histomolecular features, and imaging/radiomics markers are synthesized. Standard therapies (maximal safe resection, involved‐field radiotherapy, and alkylating chemotherapy) are reviewed in a grade‐spanning, risk‐adapted framework. Therapeutic advances are highlighted, with particular emphasis on brain‐penetrant IDH inhibition (vorasidenib) and on emerging strategies including vaccines, checkpoint combinations, epigenetic modulation, metabolic and microenvironment targeting, and novel delivery platforms. Mechanisms of resistance and recurrence, including therapy‐driven hypermutation and clonal evolution, are discussed alongside practical salvage considerations. Finally, future directions in trial design, survivorship‐oriented endpoints, and biomarker‐driven monitoring are outlined. A trajectory‐based paradigm is emphasized in which neurocognitive preservation, time to radiotherapy or chemotherapy, and patient‐reported outcomes are prioritized while durable disease control is pursued across decades‐long survivorship.

## Graphical abstract

This graphical abstract synthesizes the core logic of our revised review by linking integrated diagnosis, risk stratification, and trajectory‐based management in IDH‐mutant astrocytomas across CNS WHO Grades 2–4. It highlights lineage‐defining molecular classification (IDH mutation with core co‐alterations), integrated histomolecular grading, and key adverse biomarkers (including CDKN2A/B homozygous deletion defining Grade 4), together with imaging and radiomics to quantify tumor burden and refine prognosis. These multidimensional variables converge into an integrated risk framework that informs initial and longitudinal decision‐making. The diagram maps risk strata to management trajectories—maximal safe resection as the cornerstone, followed by risk‐adapted chemoradiation for higher grade disease and IDH inhibition strategies that can defer chemoradiation in selected Grade 2 patients—while positioning emerging modalities (immunotherapy, epigenetic, and metabolic targeting) and novel delivery technologies as future directions. Overall, the schematic emphasizes personalized, trial‐minded care that balances durable tumor control with preservation of long‐term neurocognitive function.

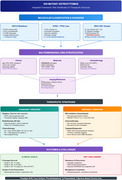

## 1 Introduction

Diffuse astrocytomas represent a major subset of infiltrative primary brain tumors in adults, characterized by biological heterogeneity, diffuse invasion, and an ultimately incurable course despite often prolonged survival [1, 2]. The discovery of recurrent isocitrate dehydrogenase (IDH) 1 and 2 mutations fundamentally reshaped glioma taxonomy, separating IDH‐mutant astrocytomas from IDH‐wildtype glioblastomas and establishing IDH status as a central determinant of prognosis and treatment strategy [2]. IDH‐mutant astrocytomas typically arise in younger adults, follow a more indolent yet chronically progressive trajectory, and require long‐term management that balances disease control with preservation of neurocognitive function and quality of life.

The fifth edition of the World Health Organization (WHO) classification recognizes IDH‐mutant astrocytomas as a distinct entity encompassing Grades 2–4, with grading based on integrated histologic and molecular features [2]. Grade 2 tumors show diffuse infiltration with mild cellularity and nuclear atypia, achieving median survivals approaching a decade [3]. Grade 3 tumors exhibit increased cellularity, cytologic atypia, and mitotic activity. Grade 4 IDH‐mutant astrocytomas are defined by either high‐grade histologic features or homozygous CDKN2A/B deletion—a molecular marker of poor prognosis that overrides conventional microscopic criteria [4, 5, 6]. Yet even within these refined WHO grades, outcomes remain heterogeneous, with overlapping survival distributions between Grades 2 and 3 and substantial variability among Grade 4 tumors [3, 6]. These observations underscore the need for multidimensional risk stratification incorporating clinical, histopathologic, molecular, and imaging‐derived factors.

Historically, landmark glioma trials enrolled molecularly unselected cohorts, frequently combining astrocytic and oligodendroglial tumors and conflating IDH‐mutant and IDH‐wildtype diseases [2]. Reinterpretation of these studies alongside emerging prospective data has reshaped the evidence base for surgery, radiotherapy, and chemotherapy in IDH‐mutant astrocytomas. The recent clinical success of brain‐penetrant IDH inhibitors in Grade 2 gliomas—providing the first targeted therapy with meaningful progression‐free survival benefit—has catalyzed broader therapeutic innovation [7]. Parallel advances in radiomics, tumor microenvironment biology, epigenetic and metabolic reprogramming, and resistance mechanisms are further redefining risk conceptualization and treatment sequencing across the disease course.

Existing guidelines have primarily focused on standard radiotherapy and chemotherapy regimens or individual advances such as IDH inhibition. While valuable, these resources often rely on the historical “low‐grade versus high‐grade” dichotomy, provide limited granularity on integrated risk models spanning WHO Grades 2–4, and offer minimal coverage of emerging strategies including immunotherapy, epigenetic modulation, metabolic targeting, tumor‐treating fields, and novel delivery platforms. The rapidly expanding preclinical and early‐phase clinical literature remains fragmented, challenging clinicians to connect mechanistic insights with practical treatment decisions.

In this review, we synthesize current knowledge on IDH‐mutant astrocytomas through the dual lens of risk stratification and therapeutic advance. We first summarize contemporary concepts in molecular classification and diagnostics, emphasizing how histopathology, co‐mutational patterns, and advanced imaging define biologically meaningful subgroups. We then integrate clinical, histologic, molecular, and radiomic markers into pragmatic risk categories across WHO grades and discuss how these inform standard surgical, radiotherapeutic, and chemotherapeutic approaches. Building on this foundation, we critically appraise emerging targeted and immunomodulatory therapies, epigenetic and metabolic interventions, microenvironment‐directed strategies, tumor‐treating fields, and innovative delivery systems. Finally, we explore resistance mechanisms and disease evolution at recurrence, outlining key future directions toward personalized, risk‐adapted management of IDH‐mutant astrocytomas.

## 2 Search Strategy and Study Selection

This narrative review provides an expert synthesis integrating clinical, radiologic, pathologic, molecular, and translational evidence relevant to risk stratification and therapeutic advances in IDH‐mutant astrocytomas across CNS WHO Grades 2–4. We performed a structured literature search from January 2000 to October 2025.

### 2.1 Data Sources and Eligibility Criteria

We searched PubMed/MEDLINE, Embase, and Web of Science for English‐language articles, and screened ClinicalTrials.gov, the EU Clinical Trials Register, major meeting abstracts (ASCO, ESMO, SNO, ASTRO), and key guidelines (WHO CNS 2016/2021, EANO, NCCN, ASCO–SNO) for practice context and backward citation tracking. Search terms combined controlled vocabulary and free text, including “IDH‐mutant astrocytoma/glioma,” “CDKN2A/B deletion,” “risk stratification,” “vorasidenib,” “radiotherapy,” “temozolomide,” “immunotherapy,” and “tumor microenvironment,” linked with Boolean operators.

We prioritized adult IDH‐mutant diffuse astrocytomas classified under WHO 2016 or 2021 criteria. Eligible evidence included randomized trials, prospective/retrospective cohorts, pooled analyses, and large institutional series reporting outcomes or treatment effects. Translational and preclinical studies were included when they provided clinically actionable insights into biology, response, or resistance. We excluded purely pediatric series, studies limited to IDH‐wildtype or non‐astrocytic entities (unless essential for comparison), and isolated case reports.

### 2.2 Study Selection and Evidence Appraisal

Titles/abstracts were screened for relevance, followed by full‐text review and assignment to prespecified domains (classification, risk stratification, standard therapies, targeted/immunologic strategies, resistance/recurrence). For overlapping cohorts, the most recent and comprehensive report was prioritized.

Given the heterogeneity of included evidence, no formal risk‐of‐bias scoring was performed. Instead, evidence was weighted qualitatively by design, sample size, and follow‐up maturity, with randomized trials, large prospective studies, and consensus guidelines serving as anchors, and retrospective series/early‐phase trials used to refine risk concepts and generate hypotheses. Recent evidence—particularly from the past decade and the most recent 3 years—was emphasized to reflect the rapidly evolving field.

## 3 Molecular Classification and Diagnostic Principles

**FIGURE 1:**
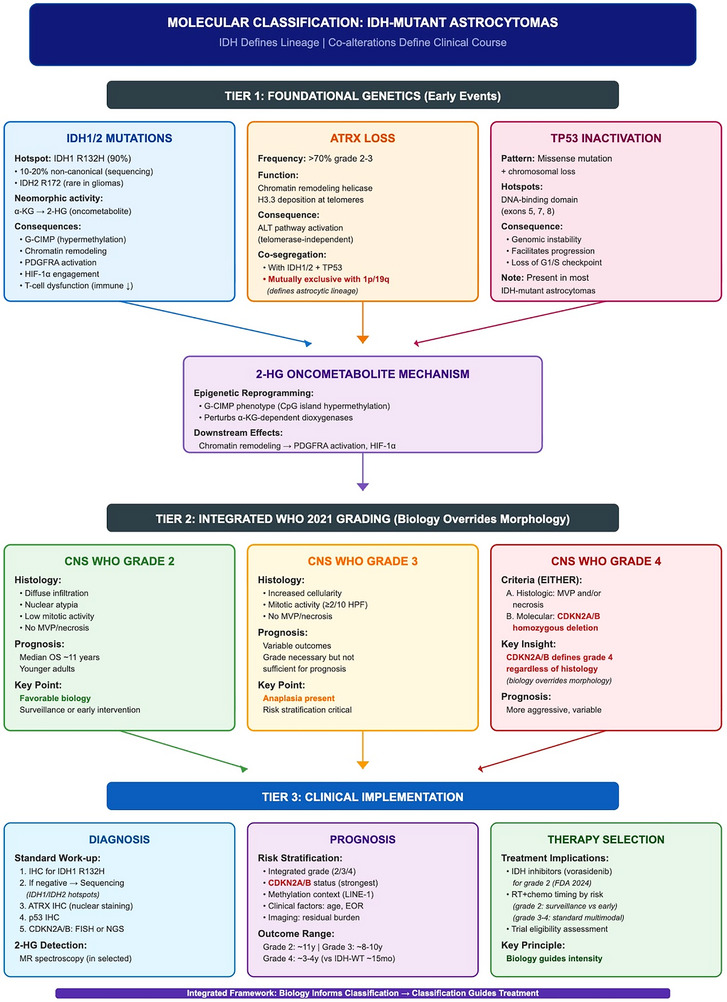
Integrated molecular classification and diagnostic principles in IDH‐mutant astrocytoma. Schematic overview of the core molecular hallmarks of IDH‐mutant astrocytoma—IDH1/2 mutation (2‐hydroxyglutarate production), ATRX loss (alternative lengthening of telomeres activation), and TP53 inactivation (genomic instability)—converging on an early lineage/epigenetic program that underpins tumor biology. Histopathology together with molecular features informs CNS WHO grading (Grades 2 and 3 with increased mitotic activity, and Grade 4 defined by CDKN2A/B homozygous deletion) and supports an integrated molecular classification framework. This integrated approach enables downstream risk stratification and treatment selection, advancing personalized neuro‐oncology. 2‐HG, 2‐hydroxyglutarate; ALT, alternative lengthening of telomeres; ATRX, alpha thalassemia/mental retardation syndrome X‐linked; CNS WHO, World Health Organization classification of tumors of the central nervous system; IDH, isocitrate dehydrogenase; TP53, tumor protein p53.

IDH defines the lineage; co‐alterations and late events define the clinical course. As summarized in Figure 1, IDH1/2 mutation with ATRX loss and TP53 inactivation establishes the early astrocytoma lineage program, while later events (including CDKN2A/B homozygous deletion) help define integrated CNS WHO Grades 2–4 and inform risk stratification and treatment selection.

### 3.1 Foundational Genetics of IDH‐Mutant Astrocytomas

High‐grade gliomas likely arise from neural precursor populations; accumulating evidence suggests that astrocyte‐like neural stem cells in the subventricular zone (SVZ), carrying low‐level somatic mutations, may serve as cells of origin [8, 9, 10, 11, 12, 13]. These tumors harbor multipotent glioma stem‐like cells with self‐renewal and multilineage differentiation capacity, enabling repopulation and contributing to therapeutic resistance when this compartment is not eradicated [14, 15, 16, 17]. Integrative genomic and epigenomic studies, together with genetically engineered models, have clarified key molecular drivers of diffuse gliomas while underscoring intratumoral heterogeneity that shapes subtype specification and treatment sensitivity [9, 10, 11, 12, 18, 19, 20, 21, 22, 23]. At a mechanistic level, diffuse glioma initiation and progression reflect oncogene activation, tumor suppressor inactivation, apoptotic evasion, DNA repair dysregulation, and extensive epigenetic reprogramming [9, 24].

In WHO Grade 2 IDH‐mutant astrocytomas, three recurrent, early cooperating alterations form the canonical genetic backbone: IDH1/2 mutation, ATRX inactivation, and TP53 loss.

#### 3.1.1 IDH Mutations

Somatic mutations affecting the Krebs cycle enzyme IDH1—predominantly at the R132 residue—were first identified in 12% of glioblastoma samples in 2008 and are now recognized as early, lineage‐defining events in diffuse gliomagenesis, occurring more commonly in lower grade gliomas [25, 26, 27]. These mutations confer neomorphic enzyme activity that produces the oncometabolite R(–)‐2‐hydroxyglutarate (2‐HG), driving a CpG island methylator phenotype through epigenetic dysregulation [28, 29, 30, 31]. The resulting hypermethylation perturbs chromatin organization and can promote aberrant activation of oncogenic programs such as PDGFRA [32]. In parallel, 2‐HG may modulate the immune microenvironment via paracrine effects on tumor‐infiltrating T cells, potentially impairing antitumor immunity [33]. Mutant IDH1 has also been proposed to exert dominant‐negative effects on wild‐type IDH1, engaging HIF‐1α pathways that favor tumorigenesis [34]. Collectively, these mechanisms link a single metabolic lesion to coordinated remodeling of metabolism, epigenetic state, and immunity.

By definition, IDH1/2 mutations are present in IDH‐mutant astrocytomas and oligodendrogliomas and are associated with substantially prolonged survival compared with IDH‐wildtype tumors [2, 35, 36, 37, 38, 39]. IDH status is indispensable for integrated classification and can aid in distinguishing diffuse astrocytoma from reactive gliosis in limited biopsies. The clinical benefit of brain‐penetrant IDH inhibitors in IDH‐mutant low‐grade gliomas further underscores the therapeutic relevance of this pathway.

#### 3.1.2 ATRX Mutations

ATRX alterations occur in > 70% of Grades 2 and 3 IDH‐mutant astrocytomas [26, 40, 41, 42]. ATRX encodes a chromatin remodeling helicase involved in histone H3.3 deposition at telomeres; loss‐of‐function is closely linked to alternative lengthening of telomeres (ALT), enabling telomere maintenance independent of telomerase [43]. ATRX mutations strongly co‐segregate with IDH1/2 and TP53 alterations and are typically mutually exclusive with 1p/19q codeletion, a defining feature of oligodendroglioma [11, 40, 41, 42].

#### 3.1.3 TP53 Mutations

TP53 inactivation—often via missense mutation with accompanying chromosomal loss—is present in most IDH‐mutant astrocytomas [10, 24, 26, 43]. Mutations frequently involve the DNA‐binding domain (Exons 5, 7, and 8), disrupting sequence‐specific binding and core tumor‐suppressor functions [24]. TP53 loss promotes genomic instability and facilitates the acquisition of additional alterations that drive progression [24].

Together, IDH mutation + ATRX loss + TP53 inactivation define the early astrocytoma lineage program, upon which later events accumulate to govern malignant transformation, treatment sensitivity, and clinical trajectory [2, 9, 10, 11, 12, 24, 25, 26, 27, 35, 40, 41, 42, 43, 44].

### 3.2 Historical Evolution and the WHO Integrated Framework

Glioma classification has evolved from morphologic schemes to the modern WHO Classification, most recently updated in 2021 [1, 2, 8, 45, 46, 47, 48, 49]. Since 2016, diffuse gliomas have been classified using an integrated approach that combines histopathology with molecular parameters—centrally, IDH mutation status—incorporating iterative updates from cIMPACT‐NOW [1, 2, 50, 51, 52, 53, 54, 55, 56]. Current guidelines define three major adult‐type diffuse glioma entities [1, 2, 8, 57]:

1. Astrocytoma, IDH‐mutant
2. Oligodendroglioma, IDH‐mutant and 1p/19q‐codeleted
3. Glioblastoma, IDH‐wildtype

This integrated framework is not merely taxonomic: it aligns diagnosis with biology, prognosis, and increasingly, therapy selection.

### 3.3 IDH Mutations and Diagnostic Work‐Up

IDH1/2 mutations define the majority of WHO Grades 2 and 3 diffuse gliomas and confer improved prognosis [3, 27, 58]. Immunohistochemistry (IHC) for IDH1 R132H is the standard first‐line test [59, 60, 61]. However, 10%–20% of cases harbor noncanonical IDH mutations detectable only by sequencing, particularly in younger patients and in oligodendrogliomas [60, 61, 62]. Notably, IDH1 R132C is enriched in Li–Fraumeni syndrome–associated gliomas, warranting consideration of germline TP53 testing when clinically appropriate [63, 64].

If IHC is negative, sequencing of IDH1/2 is recommended for Grades 2 and 3 tumors and for Grade 4 tumors in patients < 55 years [1, 2, 3, 27, 58]. In IDH‐wildtype cases, further molecular work‐up (e.g., H3 K27M/G34 alterations, EGFR amplification, TERT promoter mutation) may be required to establish specific integrated diagnoses such as glioblastoma, IDH‐wildtype [1, 2, 55]. Biologically, mutant IDH produces 2‐HG, driving epigenetic dysregulation and altered clinical behavior; 2‐HG can be monitored in vivo using magnetic resonance spectroscopy in selected contexts [3, 27, 58].

#### 3.3.1 IDH1 vs. IDH2: Shared Biology, Distinct Clinical Context

Although IDH1 and IDH2 mutations converge biologically through 2‐HG production, they are not fully interchangeable in clinical communication [1, 2, 3, 27, 58]. In diffuse gliomas, IDH1 mutations are substantially more common and are classically enriched at R132 (with R132H as the predominant hotspot), whereas IDH2 mutations are less frequent and typically involve R172 hotspots [60, 61, 62]. These differences have practical implications for diagnostic work‐up: IHC targeting IDH1 R132H captures the major subset of IDH‐mutant tumors [59, 60, 61], while sequencing is required to identify noncanonical IDH1 variants and IDH2 mutations, particularly in younger patients and selected histomolecular contexts [60, 61, 62, 63, 64]. Accordingly, integrated diagnoses should specify IDH1 versus IDH2 whenever possible, while downstream 2‐HG–driven mechanisms can be discussed jointly when evidence supports biological convergence [1, 2, 3, 27, 58].

#### 3.3.2 2‐HG–Driven Epigenetic Reprogramming and Downstream Oncogenic Pathways

IDH1/2 hotspot mutations endow the enzyme with neomorphic activity that converts α‐ketoglutarate to R(–)‐2‐hydroxyglutarate (2‐HG), creating a sustained oncometabolite state that is tightly coupled to epigenetic remodeling [28, 29, 30, 31]. Mechanistically, 2‐HG perturbs the activity of α‐ketoglutarate–dependent dioxygenases that coordinate DNA and histone demethylation programs, thereby establishing the glioma CpG island methylator phenotype (G‐CIMP) and a broadly hypermethylated chromatin landscape [30, 31]. This epigenetic “lock‐in” is thought to constrain cellular differentiation trajectories while enabling lineage‐specific oncogenic programs, providing a coherent bridge from a single metabolic lesion to stable transcriptional re‐wiring during gliomagenesis [30, 31].

Beyond global methylation shifts, IDH‐mutant gliomas exhibit higher order chromatin and regulatory architecture changes that can directly activate growth pathways [32]. In particular, insulator dysfunction and altered enhancer–promoter communication have been linked to aberrant oncogene activation, including PDGFRA, reinforcing the concept that 2‐HG–associated epigenetic remodeling is not merely a biomarker state but a driver of actionable downstream signaling [32]. In parallel, 2‐HG can influence the tumor immune milieu through suppressive effects on antitumor T‐cell function, potentially shaping immune evasion and therapeutic responsiveness [33]. Mutant IDH1 has also been proposed to exert dominant‐negative effects on wild‐type IDH1 with downstream engagement of HIF‐1α–linked programs, further integrating metabolic, hypoxia‐response, and epigenetic axes in disease initiation and progression [34]. Collectively, these convergent mechanisms position 2‐HG as a central node that couples altered metabolism to durable epigenetic reprogramming and to downstream oncogenic and microenvironmental dependencies [30, 31, 32, 33, 34]. These molecular consequences provide the biological foundation for integrated diagnosis and grading, which combine histopathologic features with key genomic alterations in contemporary classification schemes [1, 2].

### 3.4 Histopathologic Features and Grading Criteria

Despite advances in molecular diagnostics, light microscopy remains the foundation of initial classification, and historical grading was anchored in anaplasia—mitotic activity defining Grade 3 and microvascular proliferation and/or necrosis defining Grade 4—within the context of classic astrocytic versus oligodendroglial cytomorphology [1, 2]. In the integrated era, molecular alterations can supersede histology in specific contexts. Most notably, CDKN2A/B homozygous deletion independently assigns astrocytoma, IDH‐mutant to CNS WHO Grade 4, regardless of microscopic appearance, because of its strong association with adverse outcomes [2, 3, 55, 65, 66, 67]. This redefines “Grade 4” as a biologic category—not solely a morphologic one—and directly impacts risk stratification and treatment intensity.

### 3.5 Integrated Grading (CNS WHO Grades 2–4)

Astrocytoma, IDH‐mutant is a single molecular entity stratified into three grades using integrated histologic and molecular criteria [1, 2].

#### 3.5.1 Astrocytoma, IDH‐Mutant, CNS WHO Grade 2

Diffusely infiltrative tumors with nuclear atypia but lacking significant mitotic activity, necrosis, or microvascular proliferation. They typically affect younger adults, with median survival approaching ∼11 years in contemporary cohorts [3].

#### 3.5.2 Astrocytoma, IDH‐Mutant, CNS WHO Grade 3

Characterized by increased cellularity and mitotic activity but lacking necrosis and microvascular proliferation [1, 2, 55]. Even within this category, outcomes remain variable, emphasizing that grade is necessary but not sufficient for individualized prognostication [2, 3, 6, 55].

#### 3.5.3 Astrocytoma, IDH‐Mutant, CNS WHO Grade 4

Defined either by classic histologic features of glioblastoma (necrosis and/or microvascular proliferation) or by CDKN2A/B homozygous deletion [2, 3, 55, 66, 67]. Although IDH‐mutant Grade 4 tumors often occur in younger adults and may have better outcomes than IDH‐wildtype glioblastoma, they remain clinically aggressive, with substantial heterogeneity driven by co‐alterations and tumor biology [36, 68].

These integrated criteria provide the diagnostic foundation for risk stratification models that incorporate clinical status and imaging‐derived markers to delineate prognostic subgroups across the spectrum of IDH‐mutant astrocytomas.

## 4 Risk Stratification in IDH‐Mutant Astrocytomas

IDH‐mutant astrocytomas span a wide clinical continuum—from indolent tumors compatible with decades‐long survival to aggressive CNS WHO Grade 4 disease with early malignant transformation. Risk stratification in this setting is ultimately a decision tool, designed to answer three practical questions: ① how aggressive is the tumor biology, ② how large/active is the residual disease burden, and ③ how urgently should we deploy radiotherapy/chemotherapy versus defer toxicity. Contemporary frameworks therefore integrate clinical context, histomolecular grade, biomarkers, and imaging/radiomics rather than relying on any single domain.

**FIGURE 2:**
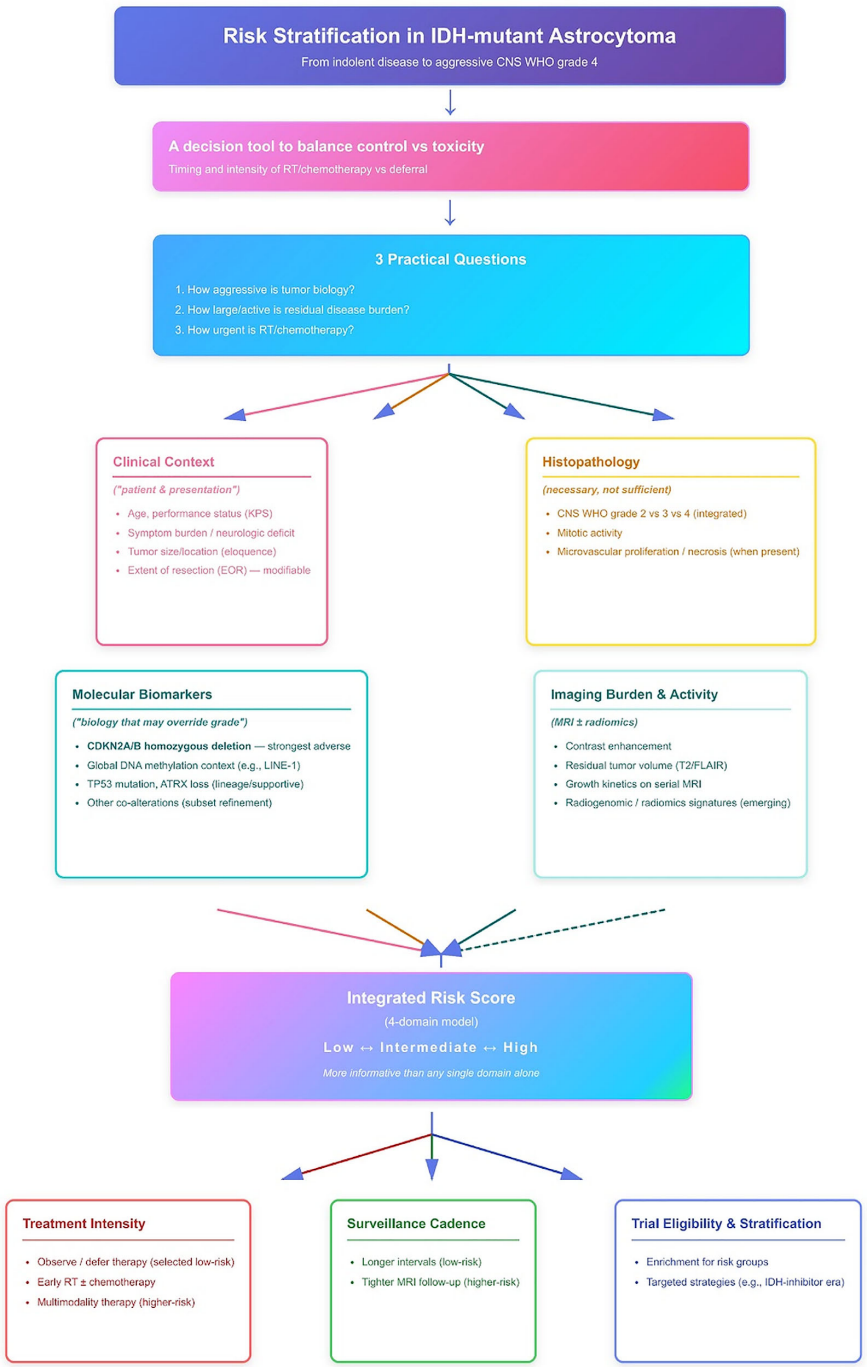
Integrated multidimensional risk stratification framework for IDH‐mutant astrocytomas. Schematic overview illustrating how clinical factors, histopathology, molecular markers, and imaging features are combined—via classical EORTC/RTOG models and emerging machine‐learning radiomics—into an integrated risk score to inform treatment selection, surveillance intensity, and clinical‐trial design. EOR, extent of resection; EORTC, European Organisation for Research and Treatment of Cancer; HPF, high‐power field; KPS, Karnofsky performance status; RT, radiotherapy; RTOG, Radiation Therapy Oncology Group.

Figure 2 summarizes a practical four‐domain model—clinical factors, histopathology, molecular biomarkers, and imaging/radiomics—that converges into an integrated risk score to guide treatment intensity, surveillance cadence, and trial stratification in IDH‐mutant astrocytomas.

### 4.1 Clinical Prognostic Factors: The “Patient and Presentation” Layer

Several clinical factors established in pre‐molecular LGG cohorts remain directionally informative in IDH‐mutant astrocytomas—age, baseline performance status, tumor size/location, neurologic deficit, and extent of resection (EOR). In the EORTC LGG analysis, age ≥ 40 years, tumor diameter ≥ 6 cm, midline crossing, astrocytic histology, and preoperative neurologic deficit each independently predicted shorter survival; accumulation of adverse factors separated prognostic groups [69]. These variables informed the pragmatic “high‐risk” definitions used in RTOG 9802 (age ≥ 40, or < 40 with incomplete resection), where adding PCV to RT improved outcomes [70]. Although developed before routine IDH testing, these EORTC/RTOG constructs remain useful for identifying clearly very‐low‐risk versus clearly high‐risk clinical phenotypes and continue to inform trial eligibility and stratification [69, 70].

Within IDH‐defined cohorts, younger age, higher KPS, seizure‐only presentation, non‐eloquent location, and more extensive resection correlate with longer PFS and OS, although the prognostic separation attributed to traditional “low‐ versus high‐grade” distinctions is attenuated once IDH status is incorporated [3, 5]. Across large histomolecular series, EOR is a consistently robust and modifiable prognostic factor: gross‐total or near‐total resection associates with delayed malignant transformation and improved survival even after adjustment for CNS WHO grade and key molecular markers [71]. Practically, this supports risk‐adapted strategies in which low symptom burden and favorable clinico‐molecular risk may justify observation or less intensive therapy, whereas multiple adverse factors favor early multimodality treatment.

### 4.2 Histopathological Grading: Necessary, but Not Sufficient

Histopathological grading remains clinically relevant but imperfect in IDH‐mutant astrocytomas. The WHO 2021 framework recognizes CNS WHO Grades 2–4 for astrocytoma, IDH‐mutant using integrated histologic and molecular criteria, including mitotic activity, microvascular proliferation, necrosis, and CDKN2A/B homozygous deletion [3, 5]. However, multiple cohorts report overlapping age at diagnosis and partially overlapping survival between Grades 2 and 3 IDH‐mutant astrocytomas, underscoring the limited discriminative power of histology alone [3]. Shirahata et al. re‐evaluated IDH‐mutant astrocytic gliomas under integrated criteria and proposed a three‐tier system with improved outcome separation compared with traditional grading [5]. Re‐analyses from CATNON materials suggest that, in tumors without CDKN2A/B homozygous deletion, mitotic count retains independent prognostic value and that a practical threshold (≥ 2 mitoses per 10 high‐power fields) associates with shorter PFS and OS [6]. Taken together, histology should be retained within risk models—but interpreted within a molecular context rather than as a stand‐alone prognostic instrument.

### 4.3 Molecular Biomarkers: The “Biology That Overrides Grade” Layer

Beyond the defining IDH1/2 mutation, several biomarkers have reproducible prognostic implications and increasingly shape integrated risk assignment.

#### 4.3.1 CDKN2A/B Homozygous Deletion

Across cohorts and an individual‐patient meta‐analysis, CDKN2A/B homozygous deletion is among the strongest adverse markers, associated with markedly reduced OS and PFS across grades [4, 71]. This evidence underpins the WHO 2021 designation of CNS WHO Grade 4 for tumors harboring CDKN2A/B homozygous deletion even in the absence of necrosis or microvascular proliferation [4, 5].

#### 4.3.2 Global DNA Methylation

Weller et al. reported that lower global DNA methylation (e.g., LINE‐1 methylation) associates strongly with Grade 4 biology and poor outcomes; combining LINE‐1 methylation with CDKN2A copy number identifies prognostic subgroups beyond histologic grade alone [71].

#### 4.3.3 TP53 and ATRX Alterations

TP53 mutation and ATRX loss support astrocytic lineage and help distinguish IDH‐mutant astrocytoma from oligodendroglioma. While typically less prognostically dominant than CDKN2A/B status, these alterations contribute to multivariable models and confirm diagnostic context [3, 5].

#### 4.3.4 IDH Mutation Type and Other Co‐Alterations

Noncanonical IDH1 and IDH2 mutations are uncommon and have been associated with slightly more favorable outcomes than canonical IDH1 R132H in some series, though findings are not fully consistent [71, 72]. Additional alterations (e.g., MGMT promoter methylation, TERT promoter mutation, copy‐number changes) may refine risk in subsets, but their independent contribution in IDH‐mutant astrocytoma is less established than in IDH‐wildtype glioblastoma [72]. Overall, these data support a histomolecular paradigm in which IDH status, CDKN2A/B copy number, and methylation context can complement—and sometimes outweigh—histology in defining clinically meaningful strata [4, 5, 71].

### 4.4 Integrated Risk Scores and Clinical Decision Frameworks: Making the Model Actionable

Recent efforts increasingly emphasize IDH‐mutant–specific multidimensional models. Re‐analyses suggest that once IDH mutation and CDKN2A/B status are incorporated, histologic grade regains prognostic relevance primarily within molecularly defined categories rather than across the entire population [3, 5]. In the Weller et al. series, a four‐group model integrating CNS WHO grade, LINE‐1 methylation, and CDKN2A/B homozygous deletion produced clearly separated survival curves and outperformed WHO grade alone [71]. Conceptually, these frameworks converge on an integrated score built from four domains:

1. Clinical context: age, KPS, symptom burden/neurologic deficit, seizure‐only presentation
2. Pathology: CNS WHO Grades 2 vs. 3 vs. 4; mitotic activity; microvascular proliferation/necrosis (where applicable)
3. Molecular biology: CDKN2A/B status; methylation context (e.g., LINE‐1); other co‐alterations where validated
4. Imaging burden/activity: enhancement pattern; residual tumor volume; growth kinetics; radiogenomic signatures

In practice, integrated assessment is used to guide decisions such as early versus deferred RT/chemotherapy in Grade 2, the intensity of multimodality therapy in Grades 3 and 4, and eligibility/stratification for trials of IDH inhibitors and other targeted approaches. Prospective validation—particularly in the post‐vorasidenib era—remains an unmet need.

### 4.5 Imaging and Radiomics: Quantifying Burden and Biological Behavior

Imaging provides structural and functional surrogates of tumor biology and is increasingly incorporated into risk stratification. In IDH‐mutant astrocytomas, conventional MRI markers reflecting aggressiveness or higher active burden include:

1. Contrast enhancement,
2. substantial postoperative residual disease / higher residual T2/FLAIR volume,
3. rapid growth on serial imaging, and
4. imaging patterns suggesting higher grade biology in context.

Across cohorts, enhancement and higher residual tumor volume correlate with shorter PFS and OS and are therefore used as pragmatic imaging risk markers alongside histomolecular assessment [71, 73].

A widely cited radiogenomic clue is the T2–FLAIR mismatch sign, defined by predominantly homogeneous T2 hyperintensity with relative FLAIR hypointensity except for a hyperintense peripheral rim. Multiple studies indicate high specificity for IDH‐mutant, 1p/19q–non‐codeleted astrocytoma, although sensitivity is limited and its prognostic value is inconsistent [72, 73, 74]. Pathologic correlation suggests an association with microcystic change in subsets [74]. Accordingly, T2–FLAIR mismatch is best viewed as a high‐specificity diagnostic enrichment sign, rather than a stand‐alone prognostic marker.

Beyond qualitative signs, radiomics and machine‐learning approaches extract quantitative MRI features (shape, texture, intensity, spatial heterogeneity) to predict molecular subtype, grade, and outcome. Systematic analyses suggest that radiomics models using multimodal MRI can predict IDH status and 1p/19q codeletion with high accuracy and may estimate survival in diffuse gliomas [75, 76]. Deep‐learning models have also shown promise for automated grading and risk prediction using multimodal MRI, supporting future integration of radiogenomic scores—particularly when tissue sampling is limited [71, 76]. At present, imaging‐based markers are most robust for treatment planning and risk enrichment (e.g., anticipating diagnosis before biopsy; estimating EOR and residual disease). As models become standardized and externally validated, imaging‐derived features are likely to be incorporated more formally into IDH‐mutant risk schemas and trial stratification.

## 5 Standard Therapies for IDH‐Mutant Astrocytomas

### 5.1 Surgical Management

Maximal safe resection is the cornerstone of initial management for suspected diffuse gliomas across grades, including IDH‐mutant astrocytomas. Surgery is both diagnostic and therapeutic, providing tissue for integrated histomolecular classification while reducing tumor burden and alleviating mass effect. The extent of resection—from gross total resection to subtotal resection or biopsy—is determined by tumor location, size, imaging characteristics, and the functional risk to eloquent cortex and critical white matter pathways. Whenever feasible, surgery should be undertaken at high‐volume centers with dedicated oncologic neurosurgical expertise to optimize outcomes [77, 78].

#### 5.1.1 Surgical Planning

Preoperative planning integrates standard structural MRI with advanced functional and connectivity imaging. Functional MRI and diffusion tensor imaging help define the relationship between tumor, eloquent cortex, and major white matter tracts, thereby informing strategies to maximize resection while preserving neurological function [79]. Intraoperatively, neuronavigation, cortical and subcortical mapping, intraoperative MRI or ultrasound, and fluorescence‐guided techniques can further refine anatomical orientation and increase the likelihood of achieving maximal safe resection with minimal injury to normal brain tissue [80].

#### 5.1.2 Timing of Surgery

Surgery is generally indicated at presentation for large, symptomatic, or radiologically aggressive tumors, including lesions with contrast enhancement, necrosis, or substantial edema. For small, nonenhancing, minimally symptomatic lesions suggestive of CNS WHO Grade 2 IDH‐mutant astrocytoma, contemporary consensus likewise favors early maximal safe resection over watchful waiting when a meaningful resection is technically and functionally achievable [77, 78]. Observational series of diffuse low‐grade gliomas consistently associate early intervention with longer survival, improved seizure control, and the added benefit of tissue acquisition for integrated diagnosis and risk stratification [77, 79, 80, 81, 82, 83, 84].

#### 5.1.3 Extent of Resection

Multiple retrospective and prospective series support an association between greater extent of resection and improved progression‐free and overall survival in diffuse gliomas, including IDH‐mutant astrocytomas [81, 85, 86, 87, 88, 89, 90]. For resectable tumors, complete or near‐complete resection is consistently superior to subtotal resection or biopsy alone, and in IDH‐mutant astrocytomas EOR remains an independent prognostic factor after adjustment for age, grade, and other clinical variables [86, 88]. Very small postoperative residual volumes (e.g., ≤ 5 cm^3^ on early postoperative MRI) have been associated with significantly worse survival compared with radiographically complete resection, underscoring that even limited residual disease can influence long‐term outcomes [88]. Taken together, these data support maximal safe resection as the foundational step in the management of IDH‐mutant astrocytomas.

### 5.2 Selection of Postoperative Therapy

Surgery alone is rarely curative for diffuse gliomas, including IDH‐mutant astrocytomas, and most patients ultimately require adjuvant radiotherapy (RT), chemotherapy, IDH‐targeted agents, or combinations thereof [91]. Postoperative management should be individualized and risk‐adapted based on tumor grade, imaging features, residual tumor burden, clinical status, and patient preference. For IDH‐mutant Grades 3 and 4 astrocytomas, consensus supports immediate postoperative RT combined with chemotherapy given their more aggressive behavior and higher risk of early progression [92]. By contrast, the optimal timing and intensity of adjuvant therapy for Grade 2 tumors remains controversial, with approaches ranging from early RT plus chemotherapy to active surveillance or upfront IDH inhibition [91, 92, 93]. Across grades, decisions should balance expected survival benefit against neurocognitive and systemic toxicity, using shared decision‐making to align treatment with patient values and life circumstances [91, 92, 93, 94, 95].

#### 5.2.1 Grade 2 IDH‐Mutant Astrocytomas

##### 5.2.1.1 Postoperative Pathways (Outside Clinical Trials)

Postoperative management for IDH‐mutant CNS WHO Grade 2 astrocytomas generally follows one of three pathways: ① active surveillance, ② radiotherapy (RT) plus chemotherapy, or ③ IDH‐targeted therapy in selected patients when the goal is to defer chemoradiation‐related neurotoxicity [96]. Vorasidenib, an oral dual IDH1/2 inhibitor, received US FDA approval in August 2024 for Grade 2 IDH‐mutant astrocytomas and oligodendrogliomas, based predominantly on the phase 3 INDIGO trial [97, 98].

##### 5.2.1.2 Risk Stratification (Pragmatic Triggers for Early Chemoradiation)

Risk stratification is increasingly anchored in clinical urgency and imaging biology, rather than age or grade alone [96, 97]. Features commonly treated as “higher risk / higher urgency” include:

1. persistent or progressive neurologic symptoms,
2. contrast enhancement on MRI,
3. substantial postoperative residual disease, and
4. rapid growth on serial imaging.

Patients with these features are typically directed toward earlier RT plus chemotherapy, whereas those without them—especially after maximal resection—may be managed with surveillance and/or IDH inhibition to defer cytotoxic therapy and preserve neurocognitive function and quality of life [96, 97].

##### 5.2.1.3 After Gross Total Resection: Surveillance as Default, Individualized Exceptions

After gross total resection, near‐term progression risk is often low and watchful waiting is commonly preferred over immediate RT/chemotherapy to limit cumulative toxicity [99]. Because INDIGO required nonenhancing measurable disease, patients without measurable residual tumor were not eligible; therefore, routine upfront IDH inhibition after complete resection remains supported by limited long‐term data [98, 100]. Decisions between surveillance and early systemic therapy should be individualized via shared decision‐making (age, comorbidities, occupation, life goals, and patient preference) [99, 100].

##### 5.2.1.4 Natural History After Complete Resection (Succinct Evidence Context)

Long‐term outcome data after complete resection largely derive from pre‐molecular series. In a prospective cohort of 111 low‐grade glioma patients with radiographically complete resection, OS at 2 and 5 years was 99% and 93%, and ∼50% remained progression‐free at 5 years; larger tumor size (≥ 4 cm), astrocytic/oligoastrocytoma histology, and residual disease (≥ 1 cm) predicted earlier recurrence [99]. Molecularly confirmed IDH‐mutant Grade 2 astrocytoma series managed initially with surveillance report 5‐year PFS of ∼30%–40%, with more extensive resection consistently associated with more favorable outcomes [38, 100]. These observations support maximal safe resection and reserving RT/chemotherapy for higher risk features or progression.

##### 5.2.1.5 Surveillance Schedule (Typical Practice)

Postoperative surveillance commonly uses contrast‐enhanced MRI every 3–4 months in the first postoperative years, extending to ∼6 monthly intervals after 1 and 2 years of stability; because most recurrences arise at or near the resection cavity margin, any new neurologic deficit, new nodular/ring‐like enhancement, or substantial edema should trigger expedited evaluation for recurrence or malignant transformation [101, 102].

##### 5.2.1.6 Management at Progression After Initial Surgery ± Observation

Management at progression is individualized based on tumor volume/location, resectability, imaging features (including enhancement), histopathology at recurrence, prior therapies, and patient priorities [101, 102]. Common components include:

1. Re‐resection (preferred when feasible)
  When radiographically and functionally resectable, re‐resection provides cytoreduction and updated tissue for grading and molecular profiling. If disease remains Grade 2 with minimal residual burden, renewed surveillance without immediate adjuvant therapy may be reasonable in carefully selected patients [101, 102].
2. IDH inhibition (vorasidenib) for selected nonenhancing, lower‐burden disease
  In low‐volume, nonenhancing progression where deferring RT and alkylating chemotherapy is prioritized, vorasidenib offers a noncytotoxic strategy for selected individuals; practical selection hinges on residual burden, growth kinetics, prior alkylator exposure, and patient priorities (notably neurocognition and long‐term treatment burden) [98, 102, 103, 104, 105].
3. Escalation for aggressive/voluminous or enhancing progression
  Large, symptomatic, rapidly progressive, or enhancing recurrences generally warrant multimodality escalation: re‐resection when feasible, followed by RT plus chemotherapy, aligned to updated pathology and molecular risk profile (Grades 2 vs. 3) [101, 102].

##### 5.2.1.7 Subtotal Resection Without Uncontrolled Symptoms: The INDIGO‐Defined Niche

In patients with residual disease after subtotal resection but without uncontrolled symptoms—particularly with nonenhancing measurable disease—vorausidenib is increasingly used to defer RT and alkylating chemotherapy [98, 103, 104, 105]. The Phase 3 INDIGO trial randomized 331 patients ≥ 12 years with residual or recurrent WHO Grade 2 IDH‐mutant gliomas (astrocytoma and oligodendroglioma) and nonenhancing measurable disease (≥ 1 cm) to vorasidenib (40 mg daily) or placebo [98]. Vorasidenib prolonged PFS (27.7 vs. 11.1 months; HR 0.39, 95% CI 0.27–0.56) and reduced the need for subsequent therapeutic intervention (85.6% vs. 47.4% free of further treatment at 18 months) [98]. The most notable toxicity was grade ≥ 3 ALT elevation (∼10%); serious adverse events were uncommon (< 2%) [98, 104].

Because INDIGO excluded enhancing tumors, current practice generally reserves vorasidenib for nonenhancing lesions in which the risk of histologic undergrading is considered low (e.g., after generous sampling) [98, 103, 104, 105]. There is no consensus residual‐size threshold; expert reviews emphasize individualized decisions best made in a multidisciplinary setting [103, 104, 105]. Other oral IDH inhibitors show activity in early‐phase studies but remain investigational in glioma, and their role relative to vorasidenib, RT, and temozolomide is undefined [104, 105, 106].

##### 5.2.1.8 Uncontrolled Symptoms or Progression on Vorasidenib: Escalation to RT Plus Chemotherapy

If patients develop uncontrolled symptoms or radiologic progression on vorasidenib, RT plus chemotherapy becomes the preferred strategy unless repeat surgery is feasible and expected to provide meaningful benefit [107].

1. Rationale for RT plus chemotherapy
  Randomized trials in diffuse low‐grade gliomas support RT combined with chemotherapy (PCV or temozolomide), particularly in high‐risk patients [107, 108, 109, 110]. RTOG 9802 randomized 251 high‐risk low‐grade glioma patients to RT (54 Gy) with or without six cycles of PCV [70, 111]. With long follow‐up, RT plus PCV improved OS (13.3 vs. 7.8 years; HR 0.59, *p* = 0.003) and prolonged PFS compared with RT alone [70]. Grades 3/4 hematologic toxicities were more frequent with PCV, without treatment‐related deaths [111]. Cognitive function assessed by MMSE was generally maintained in both arms [112]. In a molecularly annotated subset, benefit was also observed in IDH‐mutant astrocytomas and oligodendrogliomas [113]. Preliminary data from ECOG‐ACRIN E3F05 also support RT plus temozolomide in low‐grade gliomas [110]. Chemotherapy alone without RT is generally avoided in IDH‐mutant astrocytomas given less durable control when RT is omitted [114].
2. Choosing PCV versus temozolomide
  No randomized trial directly compares RT + PCV versus RT + temozolomide specifically in IDH‐mutant Grade 2 astrocytoma. In practice, temozolomide is often favored for convenience and tolerability, while PCV remains reasonable for fit patients given the survival benefit in RTOG 9802 [110, 115]. For temozolomide scheduling, many clinicians use RT followed by adjuvant temozolomide (often 12 cycles) without concurrent dosing, extrapolating from CATNON (Grade 3) where adjuvant—but not concurrent—temozolomide drove benefit; however, ECOG‐ACRIN E3F05 used both concurrent and adjuvant temozolomide, and the relative contribution of each remains uncertain [110]. RTOG 0424 (single‐arm) reported favorable long‐term outcomes versus historical controls using RT with concurrent and adjuvant temozolomide in high‐risk low‐grade glioma, though cross‐trial comparisons are limited [116, 117, 118, 119].

**FIGURE 3:**
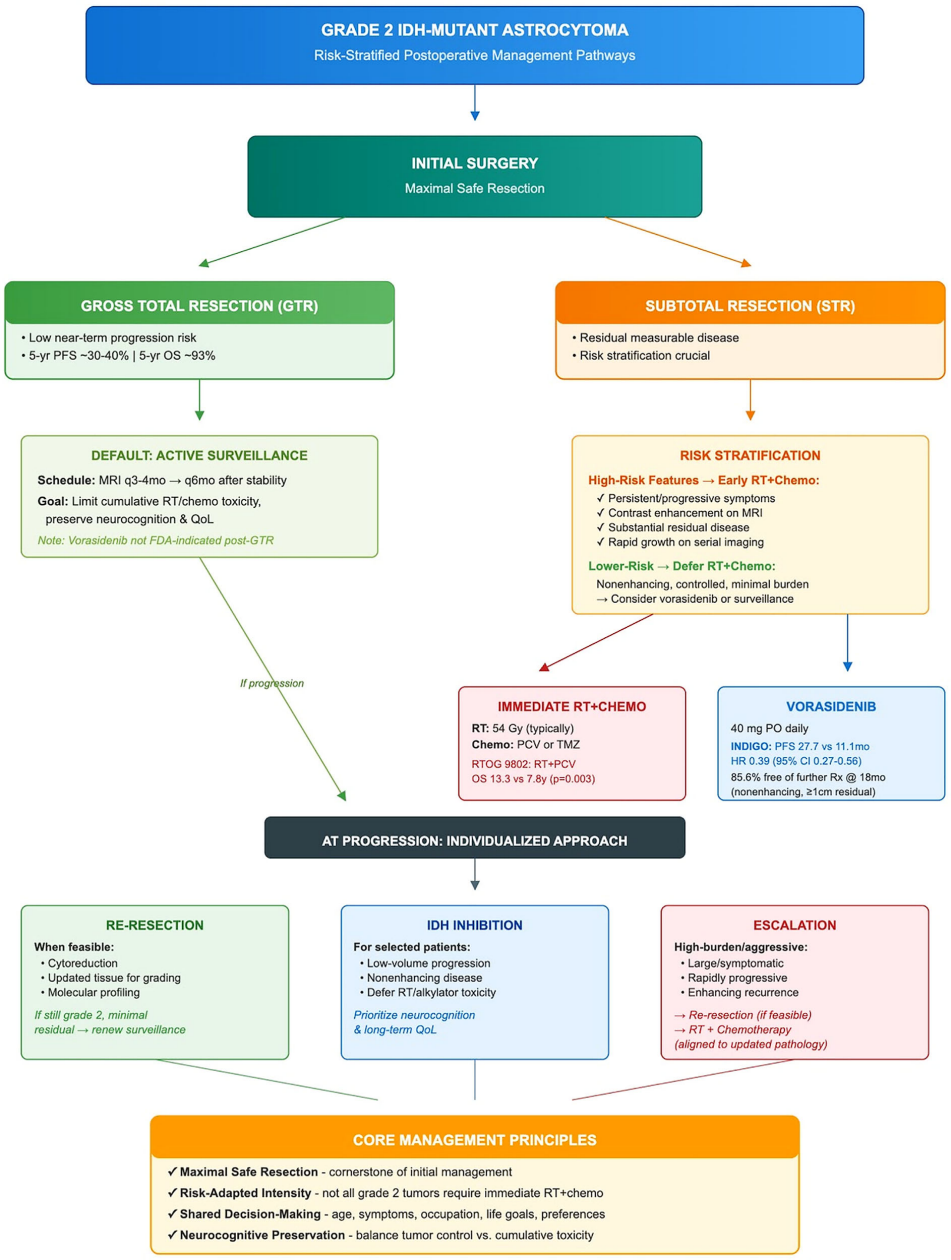
Management algorithm for WHO Grade 2 IDH‐mutant astrocytomas in the evolving molecular era. Flowchart outlining initial management after gross total resection versus subtotal resection/biopsy, subsequent risk stratification, and escalation pathways for progression or high‐risk features, including re‐resection, radiotherapy plus chemotherapy, or IDH inhibition (e.g., vorasidenib), with emphasis on active surveillance and shared decision‐making. GTR, gross total resection; MRI, magnetic resonance imaging; RT, radiotherapy; STR, subtotal resection.

Figure 3 outlines a pragmatic, risk‐triggered postoperative pathway for Grade 2 IDH‐mutant astrocytomas, linking extent of resection (GTR vs. STR/biopsy), high‐risk imaging/clinical features, and progression status to surveillance, IDH inhibition, re‐resection, or escalation to RT plus chemotherapy within a shared decision‐making framework.

#### 5.2.2 Grade 3 IDH‐Mutant Astrocytomas

##### 5.2.2.1 Selection of Therapy

For newly diagnosed IDH‐mutant CNS WHO Grade 3 astrocytomas, standard postoperative management typically consists of radiotherapy (RT) followed by adjuvant chemotherapy (temozolomide or PCV), reflecting evidence from randomized trials in 1p/19q–non‐codeleted anaplastic gliomas that demonstrated superior survival with combined‐modality therapy versus RT alone [108, 109, 119, 120].

Temozolomide is generally preferred in contemporary practice because of its favorable toxicity profile, ease of administration, and evidence base supporting RT followed by adjuvant temozolomide in this population [108, 109]. At present, IDH inhibitors (e.g., vorasidenib) have no established role in Grade 3 tumors outside clinical trials, although studies are evaluating IDH inhibition in recurrent disease and in combination regimens [100].

##### 5.2.2.2 Supporting Evidence

The EORTC 26053–22033 CATNON trial enrolled 751 adults with 1p/19q–non‐codeleted Grade 3 gliomas (including both IDH‐mutant and IDH‐wildtype tumors) and randomized patients to:

1. RT alone,
2. RT with concurrent temozolomide,
3. RT with concurrent plus adjuvant temozolomide (12 cycles), or
4. RT with adjuvant temozolomide alone [108].

At a median follow‐up of 56 months, adjuvant temozolomide significantly improved overall survival versus no adjuvant temozolomide (82 vs. 47 months; HR 0.64, 95% CI 0.52–0.79), with a pronounced benefit in IDH‐mutant tumors (117 vs. 78 months; HR 0.48, 95% CI 0.35–0.67); no benefit was observed in IDH‐wildtype tumors (21 vs. 19 months; HR 1.0) [109]. Concurrent temozolomide did not improve survival overall or within the IDH‐mutant subgroup, supporting RT followed by adjuvant temozolomide as the preferred regimen [109].

Earlier trials (EORTC 26951; RTOG 9402) in anaplastic oligodendroglioma/oligoastrocytoma populations showed survival benefit with RT plus PCV—most prominently in 1p/19q‐codeleted tumors—with only a nonsignificant trend in IDH‐mutant, non‐codeleted subsets [119, 120, 121]. Accordingly, temozolomide has largely supplanted PCV for IDH‐mutant Grade 3 astrocytomas because of better tolerability and convenience [122, 123]. Chemotherapy alone (PCV or temozolomide) without RT has not demonstrated a survival advantage in newly diagnosed Grade 2 or 3 gliomas and is generally not recommended outside clinical trials [114, 124].

**FIGURE 4:**
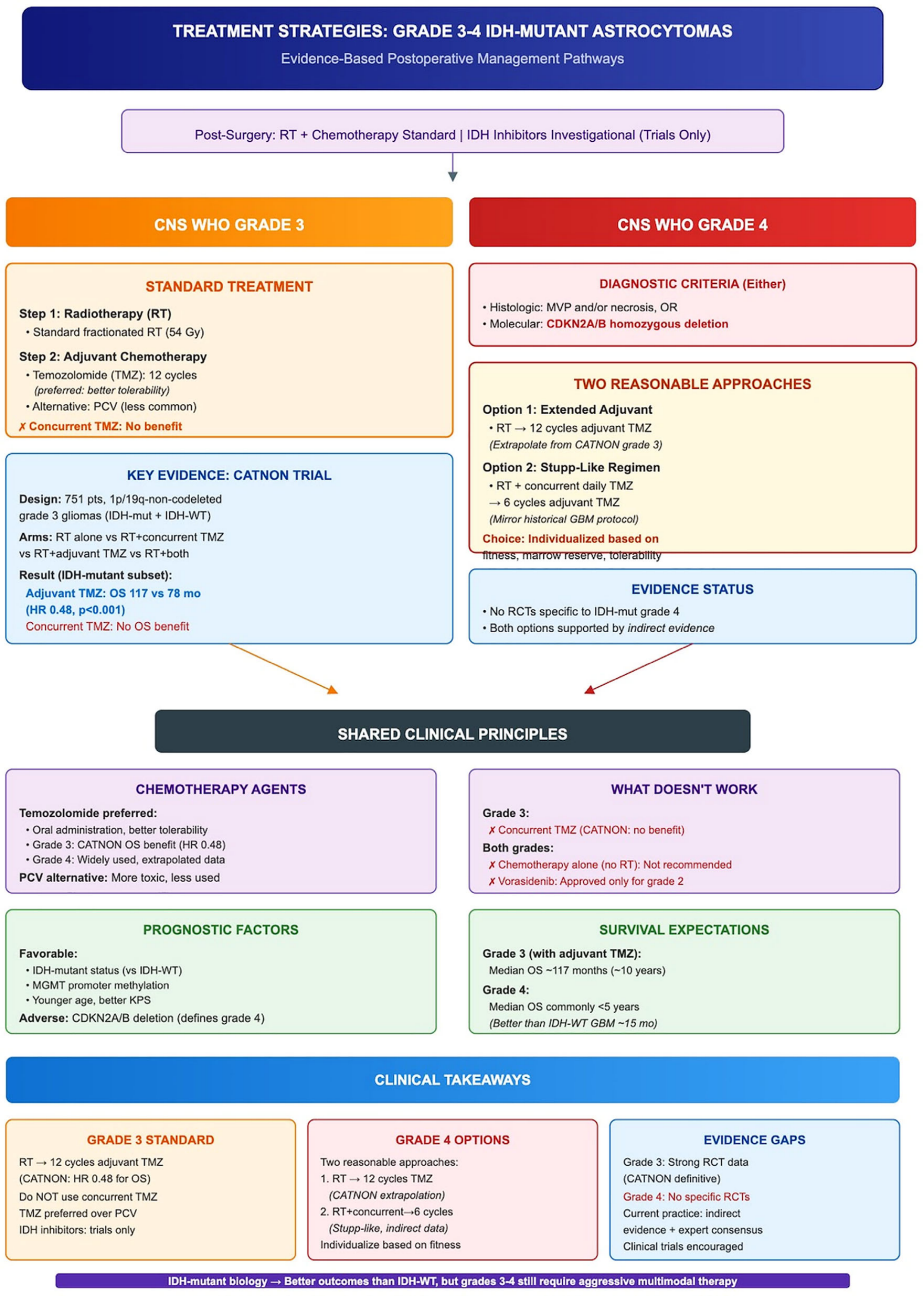
Evidence‐based postoperative management pathways for newly diagnosed CNS WHO Grades 3 and 4 IDH‐mutant astrocytomas. Schematic summarizing standard postsurgical management with radiotherapy plus alkylating chemotherapy. For CNS WHO Grade 3 tumors, the pathway reflects CATNON, supporting radiotherapy followed by adjuvant temozolomide (with no established benefit for concurrent temozolomide), and noting temozolomide is commonly preferred over PCV for tolerability. For CNS WHO Grade 4 tumors—defined by histologic features (microvascular proliferation and/or necrosis) or CDKN2A/B homozygous deletion—the figure outlines two commonly used temozolomide‐based strategies (extended adjuvant temozolomide after radiotherapy vs. a Stupp‐like regimen), acknowledging the lack of Grade 4–specific randomized evidence. IDH inhibitors and chemotherapy‐only approaches are not established outside clinical trials. CNS, central nervous system; IDH, isocitrate dehydrogenase; MVP, microvascular proliferation; PCV, procarbazine–lomustine–vincristine; RT, radiotherapy; TMZ, temozolomide; WHO, World Health Organization.

Figure 4 summarizes an evidence‐based postoperative management pathway for CNS WHO Grades 3 and 4 IDH‐mutant astrocytomas, highlighting RT followed by adjuvant alkylating chemotherapy as the backbone for Grade 3 (CATNON: benefit from adjuvant, not concurrent, temozolomide; temozolomide generally favored over PCV), and outlining Grade 4 temozolomide‐based options supported largely by indirect evidence rather than Grade 4–specific randomized trials.

#### 5.2.3 Grade 4 IDH‐Mutant Astrocytomas

Under the 2021 WHO classification, tumors previously termed “glioblastoma, IDH‐mutant” are classified as astrocytoma, IDH‐mutant, CNS WHO Grade 4 [2]. These tumors are biologically and prognostically distinct from IDH‐wildtype glioblastoma, tending to present at a younger age and more often exhibiting MGMT promoter methylation [125]. Despite comparatively more favorable outcomes, they remain clinically aggressive. Notably, CDKN2A/B homozygous deletion confers adverse prognosis and—within IDH‐mutant astrocytoma—is sufficient to assign CNS WHO Grade 4 even in the absence of microvascular proliferation or necrosis [1, 2, 4, 5]. Median survival in contemporary series commonly remains < 5 years.

In the absence of randomized trials specific to IDH‐mutant Grade 4 astrocytomas, postoperative management outside clinical trials generally follows one of two temozolomide‐based paradigms:

1. RT followed by 12 cycles of adjuvant temozolomide, extrapolating from CATNON in IDH‐mutant Grade 3 disease; or
2. RT with concurrent daily temozolomide followed by six cycles of adjuvant temozolomide, mirroring the Stupp regimen historically used for glioblastoma before routine IDH stratification.

Both approaches are regarded as reasonable by expert panels, but are supported primarily by indirect evidence rather than trials restricted to IDH‐mutant Grade 4 disease [94]. In practice, selection is individualized: clinicians favoring concurrent chemoradiation cite historical glioblastoma datasets likely containing a small IDH‐mutant subset, whereas those favoring RT followed by extended adjuvant temozolomide emphasize the CATNON signal for adjuvant benefit in IDH‐mutant, 1p/19q–non‐codeleted tumors and the biological continuum across Grades 3 and 4. Given potentially higher hematologic toxicity with concurrent temozolomide, regimen choice should incorporate patient fitness, baseline marrow reserve, comorbidities, and tolerability considerations.

### 5.3 Practical Aspects of Systemic and Local Therapies

#### 5.3.1 Vorasidenib

Vorasidenib is an oral, brain‐penetrant dual inhibitor of mutant IDH1/2 approved in August 2024 for adults and adolescents (≥ 12 years) with IDH‐mutant CNS WHO Grade 2 astrocytomas or oligodendrogliomas. The recommended dose is 40 mg once daily (20 mg once daily for patients < 40 kg), with or without food. Vorasidenib is primarily metabolized via CYP1A2; strong CYP1A2 inhibitors (e.g., ciprofloxacin) should be avoided, while CYP1A2 inducers (e.g., phenytoin) may reduce efficacy [126].

Key safety and monitoring. Hepatotoxicity is the major toxicity. In INDIGO, ALT/AST elevations of any grade occurred in 59%/46%, with Grades 3/4 elevations in 10%/5%; median time to first elevation was 57 days. Rare autoimmune hepatitis and hepatic failure were reported. Liver function testing is recommended at baseline, every 2 weeks for the first 2 months, and monthly thereafter for up to 2 years, with dose interruption/reduction as indicated [98].

*Other Adverse Events*. Hematologic effects were generally mild with low rates of Grades 3/4 events. Nonhematologic events (e.g., fatigue, seizures, musculoskeletal pain, diarrhea) were usually low grade and occurred at similar frequencies to placebo in INDIGO [98].

*Reproductive Counseling*. Animal data suggest fetal risk. Patients of childbearing potential should use effective nonhormonal contraception during treatment and for 3 months after discontinuation, as vorasidenib may reduce hormonal contraceptive efficacy; fertility considerations should be discussed before initiation [126].

Clinical takeaway: Monitor liver enzymes closely—this is the main practical constraint.

#### 5.3.2 Involved‐Field Radiotherapy

When radiotherapy is selected for IDH‐mutant astrocytomas, dose/fractionation are tailored to integrated grade: CNS WHO Grade 2 typically 45–54 Gy (1.8 Gy fractions); Grade 3, 54–59.4 Gy; Grade 4, 59.4–60 Gy (1.8–2 Gy fractions). Target volumes usually include the postoperative cavity and residual T2/FLAIR abnormality with ∼1 cm margin, using conformal planning to limit normal‐brain dose [91].

*Dose and Technique*. Trials have not shown survival benefit from dose escalation beyond conventional ranges. Hyperfractionation, proton therapy, and fractionated stereotactic radiotherapy have not demonstrated clear survival advantage over standard external‐beam RT, though they may reduce dose to critical structures in selected cases [127, 128, 129, 130, 131, 132, 133].

*Toxicities and Mitigation*. Acute effects include fatigue, alopecia, and RT‐related edema with transient neurologic worsening. Late effects include hypopituitarism (requiring endocrine surveillance), cochlear dose–related hearing loss, neurocognitive decline (often confounded by tumor course/medications), and rare secondary neoplasms typically ≥ 10 years post‐RT. IMRT/conformal planning remains central to mitigation [91].

Clinical takeaway: Optimize target volume and conformality to protect long‐term function.

#### 5.3.3 Temozolomide

Temozolomide remains the principal alkylator used with radiotherapy for IDH‐mutant astrocytomas, with schedules commonly informed by CATNON and glioblastoma experience. A standard adjuvant approach after RT is 12 monthly cycles starting ∼4 weeks post‐RT: Cycle 1, 150 mg/m^2^ orally on Days 1–5 every 28 days, with escalation to 200 mg/m^2^ for Cycles 2–12 if counts permit.

Monitoring and supportive care. CBCs are commonly checked on Days 22 and 29 of each cycle, with periodic renal function, electrolytes, and liver enzymes. Use a 5‐HT3 antagonist for antiemetic prophylaxis. Routine *Pneumocystis jirovecii* prophylaxis is generally unnecessary for adjuvant TMZ alone but may be appropriate with added immunosuppression (e.g., prolonged high‐dose glucocorticoids or concurrent RT) [108].

Clinical takeaway: Escalate only with adequate counts; reserve PCP prophylaxis for additive immunosuppression.

### 5.4 Therapeutic Advances and Targeted Agents

Therapy for IDH‐mutant astrocytomas is evolving quickly. IDH inhibition is now standard for selected Grade 2 tumors, and multiple strategies—immunotherapy platforms, epigenetic agents, metabolic interventions, and delivery technologies—are being tested for future risk‐adapted algorithms [98, 104, 106, 134]. Evidence remains heterogeneous, but the direction is toward rational combinations guided by integrated molecular and imaging risk models.

#### 5.4.1 Clinical Implications and the Emerging Landscape of IDH‐Targeted Therapy

Therapeutic decision‐making in IDH‐mutant astrocytomas is increasingly organized around risk‐adapted sequencing that integrates molecular context, imaging phenotype, and patient goals [98, 106]. Brain‐penetrant IDH inhibition has moved from proof‐of‐concept to a clinically actionable option in selected settings, supporting strategies that may defer cytotoxic therapy while preserving long‐term control [98, 102, 104, 106]. Beyond monotherapy, rational combinations and immunotherapeutic approaches (including IDH1 R132H–directed vaccine platforms) are being evaluated to deepen responses and address resistance, although efficacy data remain maturing [106, 134].

#### 5.4.2 IDH Inhibitors: From Biology to Bedside

INDIGO established vorasidenib as the first brain‐penetrant targeted agent with clinically meaningful benefit in untreated, nonenhancing Grade 2 IDH‐mutant gliomas, improving progression‐free survival and deferring RT/chemotherapy; toxicity is dominated by reversible transaminase elevation [98, 102]. Phase 1 data supported brain penetration, on‐target metabolic suppression, and durable control in recurrent disease [104]. Current consensus positions vorasidenib as a disease‐modifying option when deferring cytotoxic therapy is a core goal [98, 104, 106]. (Practical guidance is summarized in Section 5.3.1.)

Other oral IDH inhibitors (e.g., ivosidenib, enasidenib) show early radiographic/metabolic activity but remain investigational, and their role relative to vorasidenib, RT, and temozolomide is undefined [106].

Clinical takeaway: Use IDH inhibition early to defer cytotoxic therapy in appropriate patients.

#### 5.4.3 Immunotherapy: Awakening Antitumor Immunity

##### 5.4.3.1 Vaccines Targeting Mutant IDH1 and Glioma Antigens

IDH‐mutant astrocytomas often share a clonal neoantigen (commonly IDH1 R132H), enabling antigen‐specific vaccines. A Phase 1 trial of an IDH1 R132H peptide vaccine induced durable CD4^+^ T‐cell responses in most patients, with favorable safety and encouraging progression‐free survival signals [134]. Ongoing studies are testing combinations with RT, temozolomide, and checkpoint blockade, including multiepitope and dendritic‐cell platforms [106, 133].

Vaccines against broader glioma antigens (e.g., EGFRvIII, WT1, survivin) have been immunogenic but clinically inconsistent in unselected populations, supporting more molecularly defined strategies that include IDH‐mutant cohorts [135, 136, 137].

Clinical takeaway: Vaccines may fit best as combination partners anchored on clonal targets.

##### 5.4.3.2 Immune Checkpoint Inhibitors: Overcoming a Cold Microenvironment

Checkpoint blockade has shown limited efficacy in gliomas. In CheckMate 143, nivolumab did not improve overall survival versus bevacizumab in recurrent glioblastoma; subsequent PD‐1/PD‐L1 trials also reported low response rates in largely unselected cohorts [135, 136, 138].

IDH‐mutant astrocytomas add barriers—low mutational burden, low PD‐L1 expression, and a “cold” microenvironment with sparse effector T cells and enriched myeloid/regulatory populations [33, 106, 138, 139]. Preclinical data suggest 2‐hydroxyglutarate can impair T‐cell activation, supporting a mechanistic basis for primary resistance [134].

Accordingly, current approaches prioritize biomarker‐informed selection and combinations (e.g., IDH inhibition plus PD‐1 blockade; vaccines plus checkpoint inhibitors; strategies for hypermutated MMR‐deficient recurrences) [33, 106, 134, 139]. As of 2025, robust survival benefit has not been shown in IDH‐mutant astrocytomas; checkpoint inhibitors remain investigational or trial‐based in this setting [135, 136, 138].

Clinical takeaway: In IDH‐mutant disease, checkpoint inhibitors are best considered combination‐ and biomarker‐dependent.

#### 5.4.4 Cellular Therapies and Oncolytic Virotherapy

CAR T‐cell therapy shows early proof of concept in glioma. A 2024 report described marked regression in recurrent glioblastoma after intraventricular dual‐targeting CARv3‐TEAM‐E T cells, with sustained reduction beyond 150 days in one patient [140]. IL‐13Rα2–directed CAR‐T Phase 1 data support feasibility and safety, with 50% achieving stable disease or better [141, 142]. Multi‐antigen targeting (e.g., EGFR and IL13Rα2) is being pursued to address heterogeneity [143, 144].

In IDH‐mutant astrocytomas, cellular therapies remain largely preclinical; limited T‐cell infiltration suggests microenvironment remodeling or priming may be needed [106, 144, 145]. Oncolytic virotherapy is advancing: DNX‐2401 plus pembrolizumab showed acceptable safety and 52.7% 12‐month survival in recurrent glioblastoma, with modest objective responses [135, 138, 146]. These platforms are increasingly explored with checkpoint blockade or radiotherapy.

Clinical takeaway: Cellular/virotherapy is promising, but microenvironment conditioning may be prerequisite in IDH‐mutant tumors.

#### 5.4.5 Epigenetic Modulators: Reversing Oncogenic Reprogramming

IDH‐mutant gliomas show a CpG island methylator phenotype driven by 2‐hydroxyglutarate–mediated inhibition of α‐ketoglutarate–dependent dioxygenases [106, 147]. This state may create targetable vulnerabilities.

Preclinical studies suggest DNMT inhibitors (e.g., decitabine) can partially reverse hypermethylation, promote differentiation, and reduce growth [147, 148]. Combination approaches (e.g., 5‐azacytidine with temozolomide) improved outcomes in models, and trials are evaluating DNMTi monotherapy (NCT03666559, NCT03922555) [149]. Single‐cell data suggest decitabine may increase antigenicity and interferon signaling, supporting combinations with immunotherapy [150].

HDAC and histone methylation–targeting agents may enhance radiosensitivity, but toxicity and limited brain penetration have constrained development [147, 148]. No epigenetic agent is approved for glioma; IDH‐mutant–specific clinical data remain limited, but biomarker‐selected, combination‐based epigenetic modulation remains active [147, 148, 149, 150].

Clinical takeaway: Epigenetic agents are most plausible as sensitizers within rational combinations.

#### 5.4.6 Metabolic and Microenvironment‐Targeting Approaches

By diverting α‐ketoglutarate to 2‐hydroxyglutarate, IDH mutations reprogram cellular metabolism and perturb NAD^+^ homeostasis, redox balance, and DNA repair, creating putative vulnerabilities in oxidative phosphorylation, glutaminolysis, and NAD^+^ salvage that have motivated mitochondrial, glutaminase, and NAD^+^‐pathway targeting in IDH‐mutant glioma [106].

The 2‐hydroxyglutarate–shaped microenvironment also motivates combinations that reduce 2‐hydroxyglutarate, reprogram myeloid populations, restore T‐cell function, or normalize vasculature, often alongside IDH inhibition, RT, or alkylators [33, 106, 138].

Clinical takeaway: Metabolic targeting may matter most when it remodels the microenvironment for combination benefit.

#### 5.4.7 Novel Delivery Systems: Breaching the Blood–Brain Barrier

Limited BBB/BTB penetration remains a key barrier for systemic therapy. Several platforms aim to increase intratumoral exposure while limiting systemic toxicity.

*Convection‐Enhanced Delivery (CED)*. Catheter‐based positive‐pressure infusion can distribute agents through tumor interstitium. A Phase 1 study of chronic CED topotecan in recurrent glioblastoma showed acceptable safety, high intratumoral concentrations, and radiographic/metabolic activity, supporting CED for drugs, biologics, viruses, and nanoparticles [151, 152]. Data in IDH‐mutant astrocytomas are limited, but their infiltrative yet locally recurrent pattern supports consideration of focal, repeatable delivery.

MR‐guided focused ultrasound with microbubbles can transiently and spatially disrupt the BBB to increase local drug uptake; early‐phase high‐grade glioma studies suggest feasibility and short‐term safety, including improved delivery of temozolomide and antibodies [153]. For IDH‐mutant astrocytomas, such reversible BBB modulation is conceptually appealing given long survivorship and neurocognitive priorities.

Clinical takeaway: Delivery platforms may be the amplifier that converts activity into meaningful brain exposure.

Looking ahead, progress will likely depend on rational combinations delivered through optimized systemic and local strategies, guided by integrated molecular, imaging, and clinical risk stratification.

Clinical takeaway: The next gains will come from smarter sequencing and delivery, not monotherapy novelty.

## 6 Resistance Mechanisms and Recurrent Disease

Recurrence is virtually inevitable in IDH‐mutant astrocytomas. Progression reflects molecular evolution under time and treatment pressure, making longitudinal biology essential for interpreting recurrence patterns and selecting salvage strategies.

**FIGURE 5:**
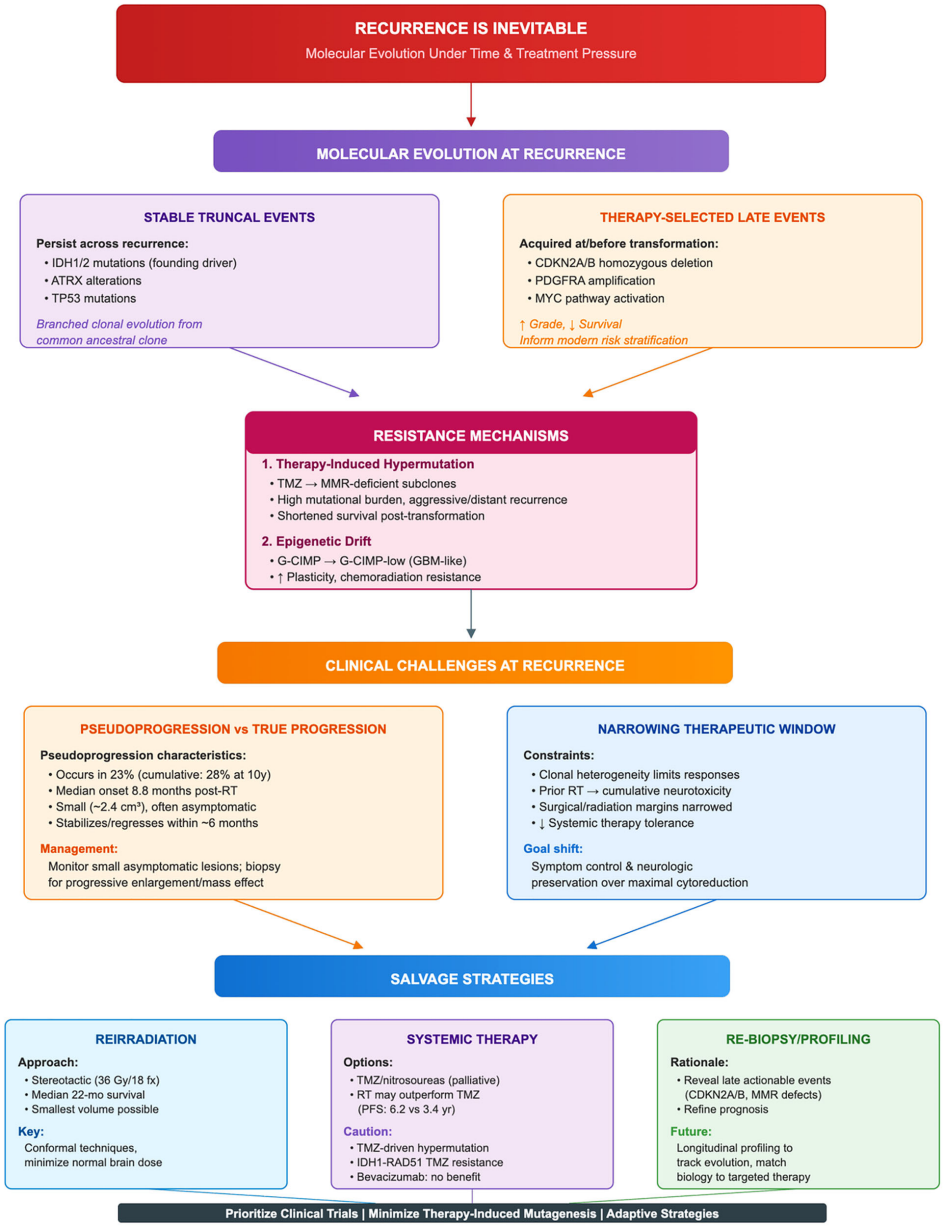
Resistance mechanisms and practical implications in recurrent IDH‐mutant astrocytomas. Conceptual schematic summarizing molecular evolution and therapy‐driven selection at recurrence (including epigenetic shifts and alkylator‐associated hypermutation), key clinical challenges in distinguishing true progression from treatment effects, and resulting implications for re‐biopsy, judicious alkylator use, and molecularly guided salvage strategies (including targeted therapies such as IDH inhibitors). G‐CIMP, glioma–CpG island methylator phenotype; MMR, mismatch repair.

Figure 5 provides an integrated schematic of how time‐ and therapy‐driven molecular evolution (including alkylator‐associated hypermutation and epigenetic drift) intersects with key clinical challenges at recurrence—distinguishing true progression from treatment effect and selecting feasible salvage options within narrowed neurotoxicity margins.

### 6.1 Molecular Evolution at Recurrence

Paired primary–recurrent sequencing reveals branched clonal evolution: early truncal events persist while therapy‐selected subclones emerge at progression [154, 155]. In IDH‐mutant astrocytomas, IDH1/2, ATRX, and TP53 alterations typically remain stable, whereas late drivers—CDKN2A/B homozygous deletion, PDGFRA amplification, and MYC‐pathway activation—are often acquired at or before malignant transformation and correlate with higher grade and worse outcomes [156, 157]. These late events increasingly inform modern risk‐stratification schemes [157].

Therapy‐induced hypermutation represents a major resistance mechanism. Temozolomide can select mismatch repair (MMR)‐deficient subclones, producing a hypermutator phenotype with high mutational burden; in IDH‐mutant lower grade gliomas, hypermutated recurrences may be aggressive and distant, significantly shortening survival after transformation [158, 159]. Epigenetically, some tumors drift from G‐CIMP toward “G‐CIMP‐low” states resembling IDH‐wildtype glioblastoma, correlating with progression, cellular plasticity, and relative chemoradiation resistance [160].

Practical implications: Re‐biopsy or re‐resection at major recurrence can reveal actionable late events such as CDKN2A/B loss or MMR defects that refine prognosis and inform trial eligibility [155, 157, 158]. Clinicians should minimize unnecessary or prolonged alkylator exposure in slowly growing tumors—particularly in younger patients—to mitigate therapy‐driven hypermutation [158, 159]. Future prognostic models will likely integrate baseline features with dynamic recurrence markers to capture evolutionary trajectories and guide adaptive treatment strategies.

### 6.2 Therapeutic Challenges and Salvage Strategies

At recurrence, clonal heterogeneity and prior local therapy constrain treatment options. Surgical and radiotherapy margins narrow, cumulative neurotoxicity limits systemic therapy tolerance, and goals often shift toward symptom control and neurologic preservation. When feasible, clinical trial enrollment should be prioritized. Standard salvage approaches—including surgery, reirradiation, and chemotherapy—require careful distinction between true progression and treatment effect, plus individualized risk–benefit assessment that considers patient age, performance status, tumor volume, and prior treatment burden.

### 6.3 Differentiating True Progression From Pseudoprogression

Pseudoprogression—new or enlarging enhancement within irradiated fields that later stabilizes or regresses—reflects treatment‐induced injury rather than viable tumor [161, 162, 163, 164, 165, 166]. In 106 radiotherapy‐treated low‐grade glioma patients, pseudoprogression occurred in 23% (median onset 8.8 months) with cumulative incidence of 13% at 1 year, 22% at 5 years, and 28% at 10 years [161, 166]. These lesions are typically small (mean ∼2.4 cm^3^), often asymptomatic, and commonly resolve within ∼6 months [161]. Vascular risk factors show inconsistent associations [167].

Modality‐specific patterns have been observed: 25% of proton‐treated patients developed multifocal edge‐of‐beam enhancing lesions (median onset ∼15 months), whereas none occurred in photon‐treated controls [168]. Because conventional MRI and amino‐acid PET may remain indeterminate, small asymptomatic lesions are often monitored closely. Biopsy or re‐resection is reserved for progressive enlargement, mass effect, or discordant clinical–radiologic evolution, while acknowledging the risk of sampling error in heterogeneous recurrent tumors.

### 6.4 Reirradiation: Balancing Benefit and Neurotoxicity

After initial full‐course radiotherapy (45–60 Gy), reirradiation is limited by cumulative neurotoxicity but can provide benefit in selected patients. In 172 recurrent glioma patients previously treated to a median of 60 Gy, stereotactic reirradiation (36 Gy in 18 fractions) was generally well tolerated, with low‐grade glioma patients achieving a median 22‐month post‐reirradiation survival [169].

Key principles: Restrict reirradiation to the smallest volume encompassing the recurrent lesion; use conformal techniques such as stereotactic approaches or intensity‐modulated radiotherapy to minimize normal brain dose; and integrate reirradiation into a comprehensive salvage plan that may include surgery and systemic therapy.

### 6.5 Systemic Therapy at Recurrence

Systemic therapy at recurrence is largely palliative, with temozolomide and nitrosoureas representing common salvage options [91, 92]. However, one retrospective analysis suggested that radiotherapy may outperform temozolomide monotherapy (PFS 6.2 vs. 3.4 years; OS 14.4 vs. 10.7 years) [170]. Pre‐molecular‐era studies reported ∼50% response rates to temozolomide in Grades 2/3 astrocytomas with meaningful 1‐year PFS in subsets [171, 172]; outcomes in higher grade recurrences have resembled relapsed anaplastic astrocytoma cohorts [173, 174]. In the TAVAREC trial, adding bevacizumab to temozolomide provided no benefit and increased toxicity in first recurrence of 1p/19q–non‐codeleted gliomas [175], leaving bevacizumab's role uncertain in this population.

Two resistance mechanisms should increasingly shape clinical practice. First, temozolomide‐driven hypermutation via MMR deficiency can lead to aggressive, enhancing, or discontiguous recurrence patterns that respond poorly to further alkylators [158, 159, 176]. Second, IDH1 mutations may confer intrinsic temozolomide resistance through RAD51‐mediated homologous recombination repair [177]. When feasible, re‐biopsy‐based molecular profiling can guide therapy selection and trial eligibility. Improving outcomes will likely require longitudinal molecular profiling to track evolutionary changes, strategies to minimize therapy‐induced mutagenesis, and matching evolving tumor biology to targeted approaches—including IDH inhibitors in the recurrent setting [97].

## 7 Prognosis

IDH‐mutant astrocytomas are responsive to multimodal therapy yet remain inherently incurable, with survival trajectories shaped by integrated histomolecular features, clinical context, and treatment approach. Molecular profiling—specifically IDH mutation status—has transformed prognostic assessment, replacing the biologically heterogeneous “diffuse astrocytoma” of the pre‐molecular era with defined entities exhibiting more predictable survival patterns.

**FIGURE 6:**
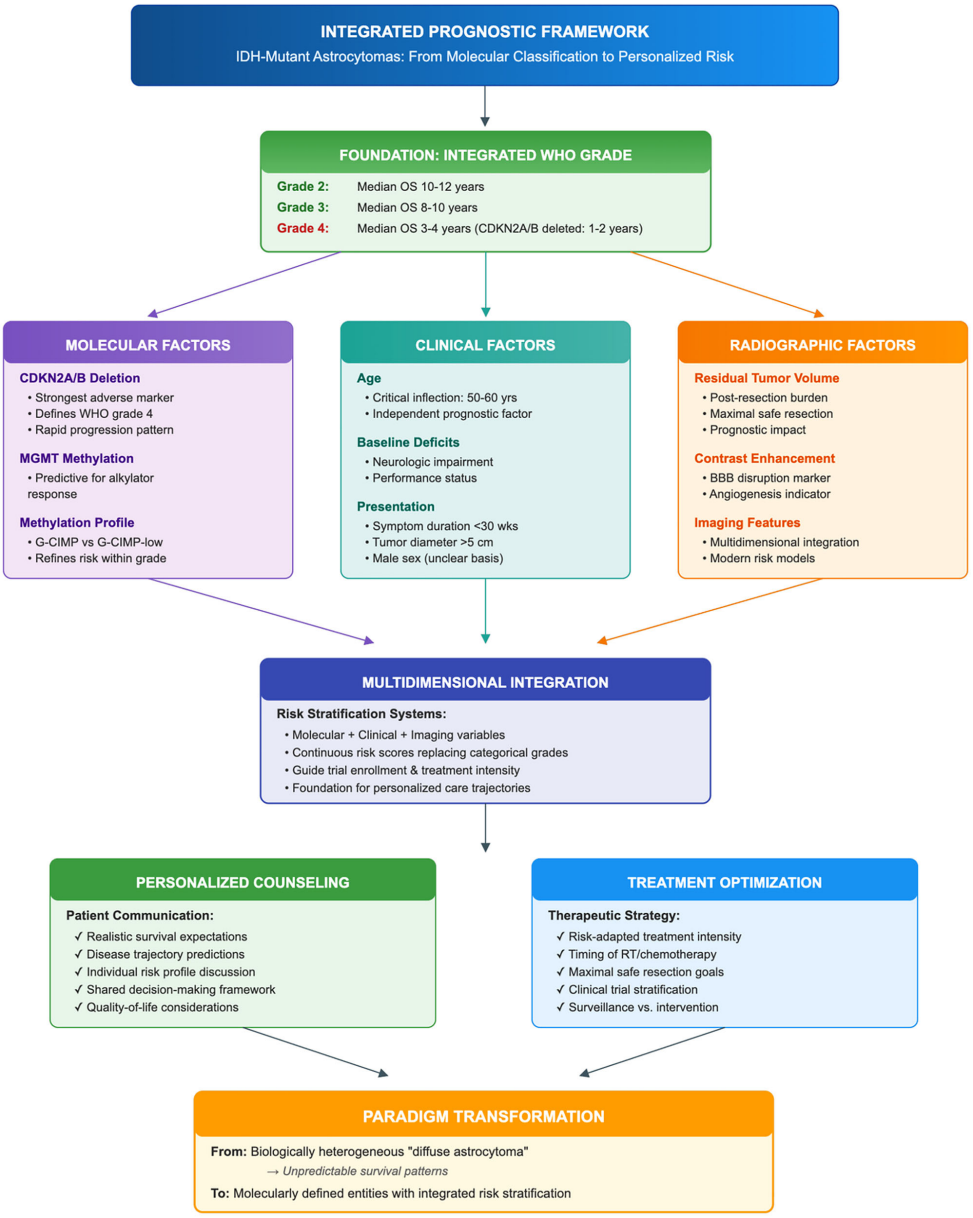
Molecularly informed prognosis in IDH‐mutant astrocytomas. Conceptual schematic illustrating how molecular profiling—centered on IDH mutation status and key modifiers such as homozygous CDKN2A/B deletion—reframes survival expectations across WHO grades and highlights additional multidimensional factors (age, symptoms, neurologic deficits, tumor size, and extent of resection/residual volume) that refine prognostic precision and support personalized therapeutic strategies.

Figure 6 summarizes how prognosis in IDH‐mutant astrocytomas is anchored by integrated WHO grade and key molecular determinants (especially CDKN2A/B homozygous deletion), and is further refined by clinical presentation and residual tumor burden to support personalized risk counseling and treatment intensity.

### 7.1 Survival by Integrated WHO Grade

For molecularly characterized IDH‐mutant astrocytomas, median overall survival correlates with integrated WHO grade, though substantial interpatient variability persists [1, 3, 5, 27, 57, 65, 178, 179, 180, 181]:

Grade 2: Median survival 10–12 years, often with prolonged radiographic and clinical stability lasting 5–10 years.

Grade 3: Median survival 8–10 years, with similarly extended disease control phases.

Grade 4: Median survival 3–4 years. Outcomes are particularly poor in tumors harboring CDKN2A/B homozygous deletion, where disease control is typically limited to 1–2 years before rapid progression [182].

These data underscore the central role of molecular diagnostics in classification, patient counseling, and treatment planning.

### 7.2 Evolving Prognostic Framework

Beyond WHO grade, prognosis reflects a complex interplay of molecular, clinical, and radiographic factors.

#### 7.2.1 Molecular Factors

CDKN2A/B homozygous deletion remains the strongest adverse marker, independently driving the aggressive behavior that justified its assignment to Grade 4 in the 2021 WHO classification [1, 3, 5, 178, 179, 180, 181]. Additional molecular features such as MGMT promoter methylation status and global methylation patterns (e.g., G‐CIMP vs. G‐CIMP‐low) contribute to refined risk stratification within each grade [71, 160].

#### 7.2.2 Clinical Factors

Historical meta‐analyses identified baseline neurologic deficits, short symptom duration (< 30 weeks), and tumor diameter > 5 cm as adverse predictors in pre‐molecular low‐grade glioma cohorts [69]. While age was not consistently independent in those analyses, recent studies in IDH‐mutant cohorts suggest age remains prognostically relevant, with the critical risk inflection point likely at 50–60 years rather than the traditional threshold of 40 years [183]. Male sex is also associated with worse outcomes, though the biological basis remains unclear [184].

#### 7.2.3 Radiographic Integration

Imaging features such as residual tumor volume and contrast enhancement are increasingly incorporated into risk models, reinforcing the prognostic value of maximal safe resection [96, 97, 185]. The integration of these multidimensional variables—clinical, molecular, and imaging—forms the foundation for modern risk‐stratification systems that guide trial enrollment and personalized treatment intensity (see Section 4).

## 8 Future Strategies and Personalized Neuro‐Oncology

IDH‐mutant astrocytomas are increasingly managed as chronic, rarely curable diseases, with survival spanning years to decades in many patients. Future progress will depend less on simply adding cytotoxic therapy and more on optimizing lifetime treatment trajectories—identifying who truly needs radiotherapy and chemotherapy, when they should be deployed, and how to sequence surgery, radiotherapy, and targeted agents to preserve long‐term neurocognitive function and quality of life.

**FIGURE 7:**
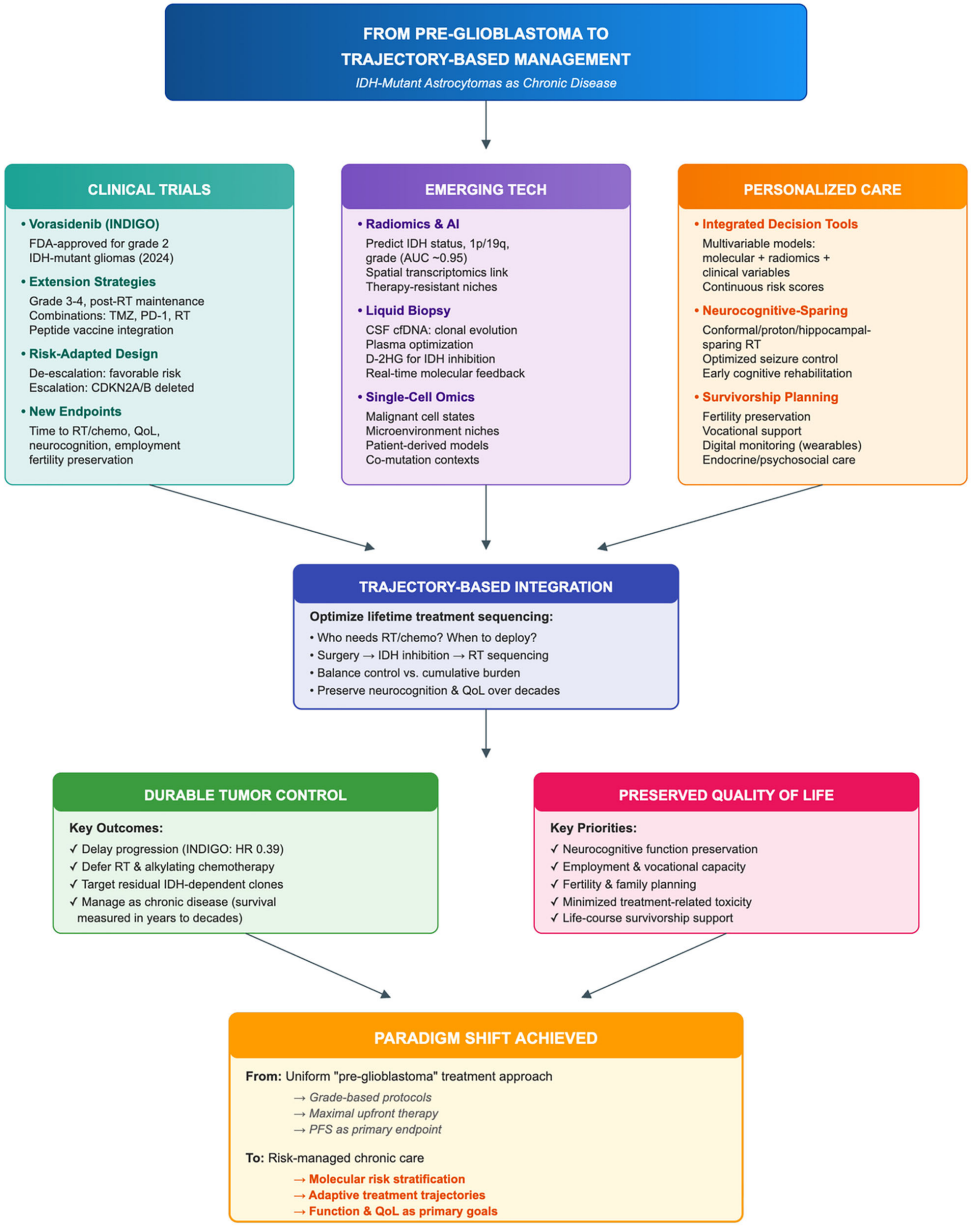
Future directions for IDH‐mutant astrocytomas: toward trajectory‐based, precision care. Overview of emerging priorities to optimize lifetime treatment trajectories, integrating targeted therapy (e.g., vorasidenib), risk‐adapted escalation/de‐escalation, next‐generation perioperative/combination trials, liquid biopsy and metabolite tracking, radiomics/AI, patient‐derived and single‐cell/spatial platforms, neurocognitive‐sparing strategies, and expanded endpoints (cognition and quality of life) to enable personalized survivorship planning.

Figure 7 outlines the emerging shift toward lifetime, trajectory‐based management—combining risk‐adapted escalation/de‐escalation (including vorasidenib in Grade 2), next‐generation perioperative and combination trials, and enabling technologies such as radiomics/AI and liquid biopsy to personalize sequencing while prioritizing neurocognitive and quality‐of‐life outcomes.

### 8.1 Clinical Trials: Redefining Endpoints and Trial Designs

The INDIGO trial established vorasidenib as the first systemic agent to significantly delay radiographic progression and the need for radiotherapy or alkylating chemotherapy in IDH‐mutant Grade 2 gliomas, validating mutant IDH as a clinically actionable target [98]. In August 2024, vorasidenib received FDA approval for patients aged ≥ 12 years with Grade 2 IDH‐mutant glioma, marking the first targeted approval for this population in over two decades [186, 187].

#### 8.1.1 Extension and Combination Strategies

Next‐generation trials are moving IDH inhibition into new settings. A perioperative Phase 1 trial demonstrated substantial brain penetration and on‐target pharmacodynamic effects for both vorasidenib and ivosidenib, with vorasidenib showing higher brain‐to‐plasma ratios and greater 2‐HG suppression, supporting feasibility of “surgical window” integration [188]. Ongoing studies are evaluating IDH inhibitors in Grades 3 and 4 disease, as post‐chemoradiation maintenance to suppress residual IDH‐dependent clones, and in rational combinations with temozolomide, PD‐1 blockade, or radiotherapy [105, 189, 190]. The VIGOR trial (NCT06809322) is evaluating vorasidenib as maintenance following standard chemoradiation [191]. In parallel, Phase 1 data on IDH1 R132H peptide vaccines suggest that neoantigen‐directed immunotherapy can be safely integrated into upfront therapy, motivating combination and risk‐adapted protocols [192].

#### 8.1.2 Risk‐Adapted De‐Escalation and Escalation

Future trials increasingly embed integrated risk models—incorporating age, performance status, grade, CDKN2A/B status, residual tumor volume, and imaging features—to test de‐escalation (e.g., IDH inhibition ± limited‐field RT in favorable‐risk patients) and escalation (e.g., intensified chemoradiation plus novel agents in CDKN2A/B‐deleted or rapidly progressive tumors). This approach recognizes that histologic grade alone inadequately captures biological heterogeneity and that molecularly defined risk strata can guide treatment intensity more precisely.

#### 8.1.3 Survivorship‐Oriented Endpoints

Beyond progression‐free survival, trial endpoints increasingly include time to first radiotherapy, time without alkylating chemotherapy, neurocognitive trajectories, patient‐reported outcomes, and functional metrics such as employment and fertility preservation. Adaptive and platform designs enrolling across CNS WHO Grades 2–4 but stratifying by molecular risk profile rather than histology alone are becoming more prominent [93, 105]. These strategies aim to shift management from a uniform “pre‐glioblastoma” mindset toward risk‐managed chronic care in which cumulative therapy burden is weighed alongside tumor control.

### 8.2 Emerging Technologies: From Deep Phenotyping to Real‐Time Monitoring

#### 8.2.1 Radiomics, Radiogenomics, and Artificial Intelligence

Quantitative MRI feature extraction coupled with machine learning enables noninvasive prediction of IDH status, 1p/19q codeletion, and WHO grade [193, 194, 195]. Multiple 2024 meta‐analyses report pooled sensitivities of 79%–88% and specificities of 80%–89% for radiomics‐based IDH prediction, with AUC values commonly 0.85–0.95; a larger synthesis across 37 studies reported pooled AUC ∼0.948, further improved by incorporating clinical variables [196, 197, 198, 199]. However, heterogeneity in pipelines, feature definitions, and validation strategies remains a major barrier, underscoring the need for prospective multicenter standardization and external validation before routine clinical deployment [193, 194, 196, 197].

Looking ahead, radiogenomic mapping may link imaging signatures to spatial transcriptomic programs and therapy‐resistant niches, enabling earlier detection of subclinical progression and more precise trial stratification [200]. As these models mature and achieve clinical‐grade reproducibility, they promise to complement tissue‐based molecular profiling with dynamic, noninvasive monitoring throughout the disease course.

#### 8.2.2 Liquid Biopsy and Metabolic Monitoring

Tumor‐derived cell‐free DNA in cerebrospinal fluid can track clonal evolution, emerging resistance, and minimal residual disease more sensitively than conventional imaging, and generally outperforms plasma for detecting glioma‐defining mutations due to blood–brain barrier constraints [201, 202, 203]. A 2024 droplet digital PCR study demonstrated plasma cfDNA detection of IDH1 R132H with concordance to tissue and correlation with overall survival, supporting feasibility in selected settings [204]. A 2025 study further suggested that increasing plasma collection volume and optimizing pre‐analytical protocols can improve circulating tumor DNA detection while maintaining specificity, potentially aiding distinction of true progression from pseudoprogression [205].

In parallel, D‐2‐hydroxyglutarate assessment by MR spectroscopy or biofluids provides a pharmacodynamic readout of IDH inhibition to guide dosing and treatment adaptation [188, 206]. As assays mature, integrated surveillance combining CSF cfDNA and metabolic imaging may become increasingly practical for patients managed with IDH inhibitors or active surveillance, offering real‐time molecular feedback without repeated surgical sampling [207, 208].

#### 8.2.3 Single‐Cell and Spatial Multi‐Omics

Single‐cell and spatial profiling reveal IDH‐mutant gliomas as interacting ecosystems of malignant cell states and microenvironmental niches, including programs linked to treatment resistance and immunosuppression. Together with patient‐derived organoids and xenografts, these platforms enable functional modeling of co‐mutational contexts (e.g., IDH mutation plus CDKN2A/B loss) and more rational prioritization of combination regimens before large trials. By dissecting cellular heterogeneity at unprecedented resolution, these technologies promise to identify targetable vulnerabilities and predict therapeutic responses with greater precision than bulk tissue analysis alone.

### 8.3 Toward Personalized, Trajectory‐Based Care

IDH‐mutant astrocytomas are well suited to precision neuro‐oncology because prognosis and treatment response reflect tractable tumor factors—IDH mutation, 1p/19q status, CDKN2A/B, MGMT methylation, imaging features, extent of resection—layered onto patient age, comorbidities, and life goals [93].

#### 8.3.1 Integrated Decision Tools

Multivariable models combining molecular markers, radiomics signatures, residual tumor volume, and clinical variables will increasingly guide treatment timing, intensity, and sequencing—including when to initiate RT/chemotherapy versus prioritize IDH inhibition. These tools aim to move beyond categorical “low‐grade versus high‐grade” frameworks toward continuous risk scores that inform individualized recommendations and enable adaptive treatment strategies as molecular and imaging profiles evolve over time.

#### 8.3.2 Neurocognitive‐Sparing Strategies

For long‐surviving patients, minimizing neurotoxicity is paramount. Approaches include tighter conformal radiotherapy planning, proton or hippocampal‐sparing RT when appropriate, dose and field de‐escalation in favorable‐risk disease, optimized seizure control, and early cognitive rehabilitation—ideally embedded as prespecified trial endpoints. As median survival approaches or exceeds a decade for many patients with Grades 2 and 3 tumors, treatment‐related cognitive decline and functional impairment rival tumor progression as determinants of quality of life, making neurocognitive preservation a central therapeutic goal.

#### 8.3.3 Life‐Course and Survivorship Planning

Personalized care will increasingly incorporate fertility preservation counseling, vocational support, driving and employment considerations, and digital monitoring through apps or wearables to track functional and cognitive trajectories in real‐world settings. Recognition of IDH‐mutant astrocytomas as chronic conditions necessitates survivorship frameworks that address the full spectrum of long‐term needs, from endocrine surveillance after radiotherapy to psychosocial support and financial planning for extended treatment courses.

Overall, the field is shifting from “Which regimen is best for this WHO grade?” toward trajectory‐based care—integrating targeted agents, deep phenotyping, and survivorship‐focused endpoints to balance durable control with preservation of function and quality of life over potentially decades of living with IDH‐mutant astrocytoma. This paradigm recognizes that optimizing outcomes requires not only controlling tumor growth but also protecting the person living with the disease.

### 8.4 Clinical Milestones and Emerging Targeted Frontiers

The therapeutic landscape of IDH‐mutant astrocytomas is undergoing a paradigm shift, transitioning from nonspecific cytotoxic approaches to biologically driven targeted interventions. To consolidate the pivotal breakthroughs—most notably the integration of brain‐penetrant IDH inhibitors—and to highlight the trajectory of ongoing clinical trials, the key milestones and emerging strategies are synthesized in Table 1.

**TABLE 1:** Recent therapeutic milestones and clinical frontiers of vorasidenib and targeted strategies in IDH‐mutant gliomas.

Clinical domain	Key evidence and major findings	References
PFS and disease control	The Phase 3 INDIGO trial demonstrated that vorasidenib significantly improved progression‐free survival (PFS) in Grade 2 tumors (median 27.7 vs. 11.1 months), reducing the risk of progression by 61%.	[98]
Treatment delay	Vorasidenib markedly delayed the time to next intervention (radiotherapy or chemotherapy) with an HR of 0.26, providing a critical window for neurocognitive preservation.	[98, 185]
Regulatory approval	Based on INDIGO data, the US FDA approved vorasidenib in August 2024 for adult and pediatric patients with Grade 2 IDH‐mutant glioma.	[186, 187]
Biological impact	Perioperative data confirmed high blood‐brain barrier penetration, > 90% reduction in 2‐HG levels, and induction of tumor cell differentiation.	[188]
Immunotherapy synergy	Clinical exploration of vorasidenib combined with pembrolizumab (PD‐1 inhibitor) is underway to evaluate synergistic metabolic‐immunologic effects.	[189]
Combination with chemo	New Phase 1b/2 protocols are investigating the safety and efficacy of combining vorasidenib with temozolomide for high‐risk cases.	[190]
Maintenance strategy	The VIGOR trial (Phase 3), initiated in 2025, is evaluating vorasidenib as a maintenance therapy following standard‐of‐care treatments.	[191]
Vaccine therapy	Beyond small molecules, mutation‐specific peptide vaccines targeting IDH1 R132H have shown the ability to induce potent antitumor T‐cell responses.	[192]

### 8.5 Emerging Diagnostic Frontiers: AI‐Driven Radiomics and Liquid Biopsy

Parallel to therapeutic innovations, the diagnostic paradigm is transitioning toward non‐invasive, high‐precision modalities. As summarized in Table 2, the integration of AI‐driven radiomics and multi‐source liquid biopsy is enabling real‐time monitoring of tumor evolution and therapeutic response.

**TABLE 2:** Advanced diagnostic technologies for molecular monitoring and risk stratification.

Diagnostic modality	Technological breakthrough and clinical utility	References
Deep learning & radiomics	Advanced AI algorithms and radiomic signatures now enable highly accurate, noninvasive prediction of **IDH mutation, 1p/19q co‐deletion**, and **ATRX status** directly from standard‐of‐care MRI.	[193, 194, 196, 197, 198]
Differential diagnosis	Integration of MRI‐based radiomics with deep learning effectively distinguishes **IDH‐mutant WHO Grade 4 astrocytomas** from **IDH‐wildtype glioblastomas**, refining pre‐operative risk stratification.	[195, 199]
Imaging genomics	Spatial and temporal heterogeneity of gliomas can be decoded through imaging genomics, providing insights into the **evolutionary trajectory** and molecular landscape without repeat biopsies.	[200]
CSF liquid biopsy	Cerebrospinal fluid (CSF) cell‐free DNA (cfDNA) serves as a sensitive tool for **tracking clonal evolution**, monitoring minimal residual disease, and detecting emergent resistance mutations.	[201, 202, 207]
Plasma ctDNA tracking	Recent evidence confirms that **IDH1 mutations are detectable in plasma** ctDNA; despite lower yields than CSF, plasma monitoring correlates with survival outcomes and treatment response.	[203, 204, 205, 208]
2‐HG metabolic MRS	Magnetic resonance spectroscopy (MRS) specifically targeting the **oncometabolite 2‐HG** provides a direct “metabolic window” to monitor the enzymatic activity of IDH inhibitors in real‐time.	[206]

## 9 Conclusion

Isocitrate dehydrogenase (IDH)–mutant astrocytomas exemplify how integrated molecular classification has transformed both prognostication and treatment in diffuse gliomas, defining a distinct entity spanning CNS WHO Grades 2–4 rather than variants within a simple “low‐ versus high‐grade” framework [2]. Although outcomes are generally superior to those of IDH‐wildtype glioblastoma, survival remains highly variable, underscoring the limitations of histology alone and the need for multidimensional risk models that incorporate clinical, histologic, molecular, and imaging features [5].

Among molecular markers, homozygous CDKN2A/B deletion has emerged as the dominant adverse factor, strong enough to assign CNS WHO Grade 4 to IDH‐mutant astrocytomas irrespective of microscopic appearance [4]. Global methylation patterns, including LINE‐1 hypomethylation and G‐CIMP‐low signatures, further refine risk and, together with IDH and CDKN2A/B status, form the backbone of contemporary histomolecular schemas [71]. Classic clinical variables—performance status, tumor size and location, seizure burden, and extent of resection—still carry prognostic weight but must now be interpreted within IDH‐defined cohorts, where their effect size is attenuated and more closely tied to residual disease and growth kinetics [69].

Maximal safe resection remains the cornerstone of management across the IDH‐mutant astrocytoma spectrum, improving seizure control, delaying malignant transformation, and prolonging survival, particularly when gross‐total or near‐total resection is achievable in non‐eloquent regions [88]. Contemporary series favor early surgery over watchful waiting even for many small, nonenhancing, minimally symptomatic lesions, given consistent associations between extent of resection, long‐term outcome, and the ability to obtain tissue for integrated diagnosis and risk stratification [82]. Postoperative strategies are then tailored according to grade, residual tumor burden, radiographic features including enhancement, and clinical risk profile, balancing survival gains from radiotherapy and chemotherapy against cumulative neurocognitive and systemic toxicity [93].

For IDH‐mutant Grades 3 and 4 astrocytomas, immediate postoperative radiotherapy plus chemotherapy remains standard of care, supported by randomized data and guideline consensus, although the optimal regimen and duration continue to be refined in molecularly annotated cohorts [109]. In contrast, management of Grade 2 disease is deliberately heterogeneous: low‐risk patients after gross‐total resection can often be observed with structured surveillance, whereas those with substantial residual disease, early progression, or enhancement are considered for early chemoradiation or, increasingly, IDH‐targeted therapy [99]. The Phase 3 INDIGO trial established brain‐penetrant IDH inhibition with vorasidenib as the first systemic treatment to significantly prolong progression‐free survival and delay the need for radiotherapy or alkylating chemotherapy in IDH‐mutant Grade 2 diffuse gliomas, validating mutant IDH as an actionable driver in routine practice [98].

Beyond IDH inhibition, multiple emerging strategies target the metabolic, epigenetic, and microenvironmental hallmarks of IDH‐mutant astrocytomas. Aberrant 2‐hydroxyglutarate–driven methylation and NAD^+^ redox imbalance provide a rationale for DNA‐methyltransferase and histone‐modifying agents, as well as inhibitors of glutaminolysis and NAD^+^ salvage pathways, although brain penetration and toxicity remain key challenges [147]. The immunologically “cold” microenvironment—characterized by low mutational burden, impaired T‐cell function, and myeloid‐dominated infiltrates—helps explain the limited efficacy of checkpoint blockade to date and motivates rational combinations of IDH inhibitors, vaccines, and immunomodulators in biomarker‐selected patients [134]. Innovative delivery platforms such as convection‐enhanced delivery and focused ultrasound–mediated blood–brain barrier opening are being exploited to increase intratumoral drug concentrations while limiting systemic toxicity, and may be particularly valuable for targeted and immune‐based agents in diffusely infiltrative disease [151].

At the same time, advances in radiomics, radiogenomics, liquid biopsy, and single‐cell and spatial multi‐omics are enabling “deep phenotyping” that captures tumor biology far beyond conventional histopathology, with the promise of non‐invasive monitoring of molecular status, minimal residual disease, and emergent resistance [196]. Looking ahead, the central challenge is to integrate these insights into pragmatic, trajectory‐based care pathways that treat IDH‐mutant astrocytomas as chronic, risk‐stratified diseases rather than inevitable precursors of glioblastoma [97]. Future practice will rely on validated multidimensional scores that combine clinical, histologic, molecular, and imaging markers to choose the least toxic, biologically rational sequence of surgery, radiotherapy, chemotherapy, and targeted agents for each individual patient, while explicitly incorporating neurocognition, survivorship, and life‐course priorities into therapeutic decision‐making [93].

## Author Contributions

All authors contributed to the conception and design of the review, literature retrieval, critical synthesis and interpretation of the evidence, and drafting and revision of the manuscript. All authors read and approved the final manuscript and agree to be accountable for all aspects of the work.

## Funding

This work was supported by departmental research funding from the Department of Neurosurgery, Yangpu District Shidong Hospital, Shanghai, China (Grant No. 12‐002‐2025).

## Ethics Statement

The authors have nothing to report.

## Conflicts of Interest

The authors declare no conflicts of interest.

## Data Availability

The authors have nothing to report.
